# Climate and the latitudinal limits of subtropical reef development

**DOI:** 10.1038/s41598-021-87883-8

**Published:** 2021-06-22

**Authors:** Lauren T. Toth, William F. Precht, Alexander B. Modys, Anastasios Stathakopoulos, Martha L. Robbart, J. Harold Hudson, Anton E. Oleinik, Bernhard M. Riegl, Eugene A. Shinn, Richard B. Aronson

**Affiliations:** 1 U.S. Geological Survey St. Petersburg Coastal and Marine Science Center, St. Petersburg, FL USA; 2 Marine and Coastal Programs, Dial Cordy & Associates, Inc., Miami, FL USA; 3 Department of Geosciences, Florida Atlantic University, Boca Raton, FL USA; 4 Independent Consultant, Glenmont, NY USA; 5 Reef Tech, Miami, FL USA; 6 Department of Marine and Environmental Sciences, Nova Southeastern University, Dania Beach, FL USA; 7 College of Marine Science, University of South Florida, St. Petersburg, FL 33701 USA; 8 Department of Ocean Engineering and Marine Sciences, Florida Institute of Technology, Melbourne, FL USA

**Keywords:** Palaeoecology, Marine biology, Palaeoclimate, Climate-change ecology

## Abstract

Climate plays a central role in coral-reef development, especially in marginal environments. The high-latitude reefs of southeast Florida are currently non-accreting, relict systems with low coral cover. This region also did not support the extensive Late Pleistocene reef development observed in many other locations around the world; however, there is evidence of significant reef building in southeast Florida during the Holocene. Using 146 radiometric ages from reefs extending ~ 120 km along Florida’s southeast coast, we test the hypothesis that the latitudinal extent of Holocene reef development in this region was modulated by climatic variability. We demonstrate that although sea-level changes impacted rates of reef accretion and allowed reefs to backstep inshore as new habitats were flooded, sea level was not the ultimate cause of reef demise. Instead, we conclude that climate was the primary driver of the expansion and contraction of Florida’s reefs during the Holocene. Reefs grew to 26.7° N in southeast Florida during the relatively warm, stable climate at the beginning of the Holocene Thermal Maximum (HTM) ~ 10,000 years ago, but subsequent cooling and increased frequency of winter cold fronts were associated with the equatorward contraction of reef building. By ~ 7800 years ago, actively accreting reefs only extended to 26.1° N. Reefs further contracted to 25.8° N after 5800 years ago, and by 3000 years ago reef development had terminated throughout southern Florida (24.5–26.7° N). Modern warming is unlikely to simply reverse this trend, however, because the climate of the Anthropocene will be fundamentally different from the HTM. By increasing the frequency and intensity of both warm and cold extreme-weather events, contemporary climate change will instead amplify conditions inimical to reef development in marginal reef environments such as southern Florida, making them more likely to continue to deteriorate than to resume accretion in the future.

## Introduction

### Climate and subtropical coral-reef development

Anthropogenic climate change is now considered to be the primary cause of coral-reef degradation globally^1,2^. Although the rate and magnitude of coral loss over the last 50 years may be without precedent in recent millennia^3^, climate has been a primary control on the rate, duration, and spatial extent of reef development throughout geologic history^4–10^. Coral-reef development is generally most extensive and most rapid in tropical environments, where temperatures are warm and stable^11,12^. Although recent coral-reef degradation has been driven primarily by elevated ocean temperatures^1,2^, many declines in the past have been attributed to cooling trends^4,5,9,13–16^. In environments where thermal conditions are marginal, even minor cooling has the potential to suppress or even shut down reef-building by species that evolved in the tropics^4,9,11,14,17,18^. Whereas a number of recent studies have suggested that marginal environments—high-latitude habitats, mesophotic reefs, and locations with elevated turbidity or upwelling—could serve as refugia from warming for thermally sensitive coral taxa^19–22^, most of these ecosystems do not support reef accretion at present^20^. An important question, therefore, is whether and how the response of marginal reefs to climatic trends in the past can be used to project the future development of today’s degrading reef ecosystems^6,7^ in response to anthropogenic warming trends and the impact of those recent trends on both warm and cold thermal extremes.

Climatic variability during the Holocene was moderate compared with changes over longer geologic intervals^4,5,23^. The Holocene epoch was nonetheless characterized by significant thermal variability over multidecadal to millennial timescales^23,24^. Average global temperatures peaked ~ 10–6 thousand years ago (ka)—an interval known as the Holocene Thermal Maximum (HTM)—before declining significantly over the Middle to Late Holocene^24,25^. Whereas tropical environments are buffered from the most extreme thermal variability, subtropical habitats are sensitive to broad-scale climatic oscillations^26–28^. For example, the relatively warm climate of the HTM drove temporary poleward expansions of corals in Florida^29^, the northern Gulf of Mexico^30^, and the high-latitude Pacific (e.g., in Japan^17^, China^16^, and the Tasman Sea^15^).

Florida’s subtropical reefs currently exist near the thermal minimum for reef development^9,11,31–33^, and periodic winter cold events frequently push them below this threshold^18,31,32,34,35^. As a result, contemporary reef growth throughout southern Florida is negligible^9,36^; however, recent range expansions of reef-building corals in Florida and other high-latitude locations have led to the suggestion that subtropical environments may serve as refugia for these corals in a future, warmer world^19,20,29,30^. It is unclear whether such range expansions would translate into expansions of reef-building in subtropical habitats. Here, we test the hypothesis that the latitudinal limits of reef-framework construction on Florida’s subtropical reefs were controlled primarily by climate during the Holocene. We track the changing geography of reef accretion along the northern extent of the Florida reef tract over the last ~ 10,000 years and compare the climatic drivers of Holocene and Anthropocene reef development to project the future of reef-building in marginal environments such as southern Florida.

### Reconstructing Holocene reef development on the SFCRT

**Figure 1:**
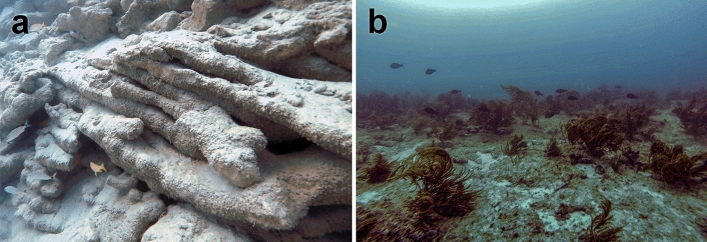
Underwater photographs of Holocene and modern reefs off Miami Beach, FL. **(a)** Holocene *Acropora palmata* reef framework on the Outer Reef in south Miami exposed by dredging in Government Cut (− 14 m mean sea level (MSL); see “Methods”). **(b)** Modern habitats in the same location dominated by octocorals, sponges, and macroalgae (− 12 m MSL). Photographs by WFP in September 2014.

The Florida Reef Tract extends more than 500 km along Florida’s Atlantic coast from Dry Tortugas National Park to northern Palm Beach County (Supplementary Fig. S1). It can be divided into two subregions based on their distinct geomorphology and geologic histories: the Florida Keys reef tract (FKRT) and the Southeast Florida Continental Reef Tract (SFCRT). Fowey Rocks reef in Biscayne National Park marks the northern extent of the FKRT (25.6° N)^31,33^, which extends southwest along the Florida Keys to the Dry Tortugas^9,37^. Whereas the FKRT has recently supported abundant populations of reef-building corals, the SFCRT to the north is a ‘relict’ reef system^38^, characterized by low-relief, hardbottom habitats with low coral cover^32,39,40^ (Fig. 1). The presence of extensive, shore-parallel reef-ridge structures or ‘terraces’ extending along much of the SFCRT^38,40,41^ is suggestive, however, of reef development during some periods of the Holocene^38^ (Fig. 1a; Supplementary Fig. S2; https://maps.ngdc.noaa.gov/viewers/bathymetry/).

**Figure 2:**
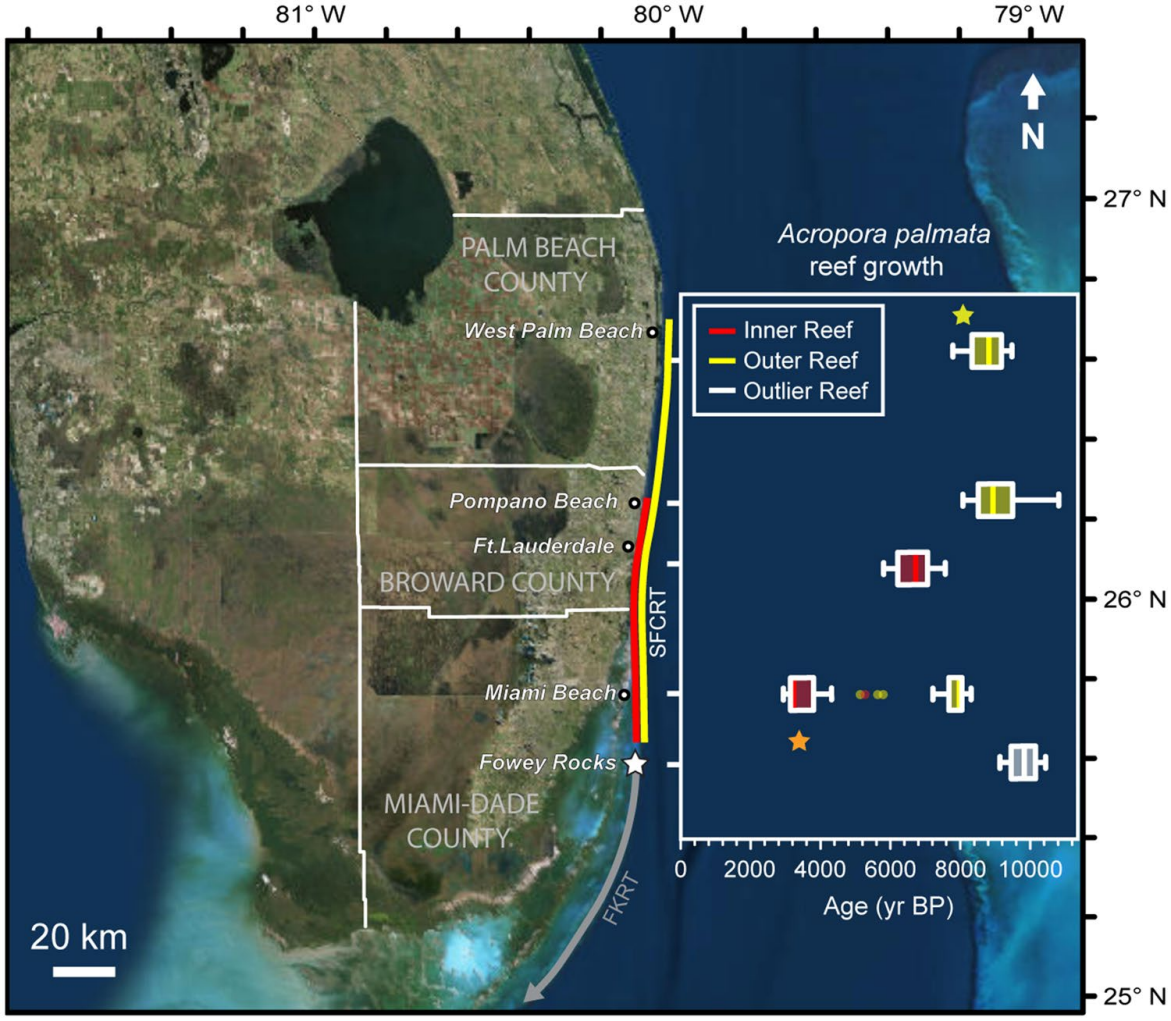
Map of the extent of the Southeast Florida Continental Reef Tract’s (SFCRT) Inner Reef (red line) and Outer Reef (yellow line) and timing of *Acropora palmata* reef growth at sampling locations (boxplots). Boxplots represent the medians (solid verticals) and interquartile ranges (boxes) of radiometric ages of *A. palmata* from each location. Error bars (whiskers) are 1.5 × the interquartile range. Points indicate data outside this range. The two stars indicate ages from *A. palmata* sampled at the northern limit (yellow, Outer Reef) and southern limit (orange, convergence of the Outer and Inner Reefs) of the Southeast Florida Continental Reef Tract. Data from the Outlier Reef at Fowey Rocks (white star), Florida Keys Reef Tract (FKRT; grey line) are also shown for comparison. Ages from the Inner Reef in Palm Beach and Miami-Dade Counties are reef-surface samples, whereas the other locations also include subsurface samples. Map image is the intellectual property of Esri and is used herein under license. Copyright 2020 Esri and its licensors. All rights reserved.

The SFCRT extends ~ 120 km along Florida’s southeast coast from just north of Fowey Rocks to northern Palm Beach County (Fig. 2, Supplementary Fig. S1). Although the continental shelf of southeast Florida is relatively narrow at just 3–4 km wide^38,40^, new mapping efforts have demonstrated that the SFCRT includes five discrete, parallel reef terraces (Supplementary Fig. S1; https://maps.ngdc.noaa.gov/viewers/bathymetry/). Listed in order of distance from shore (after Walker et al.^40^), these habitats are the Nearshore Ridge Complex (NRC; 3–5 m depth), Inner Reef (IR; ~ 8 m depth), Middle Reef (MR; ~ 15 m depth), Outer Reef (OR; ~ 16 m depth), and Deep Ridge (DR; > 25 m depth). Whereas all five reef habitats are present south of Hillsboro Inlet, the IR is absent north of the inlet, the MR terminates in southern Palm Beach County, and the OR only extends to central Palm Beach County^40^ (Supplementary Fig. S1). (Note Banks et al.^38^ suggested that the IR may actually extend slightly north of the Hillsboro inlet, as its northern limit could be obscured by seaward progradation of Florida’s shoreline.) The DR, which is a low-relief, hardbottom habitat rather than a true reef terrace, extends the furthest north, terminating near the boundary between Palm Beach and Martin Counties^40^. The distance between these habitats also varies with latitude, with the IR, MR, and OR converging in south Miami, just north of Fowey Rocks. The best developed habitats on the SFCRT are the OR and IR^38,42^, which are the focus of this study (Fig. 2). Previous geological characterizations of those reef ridges off Broward County suggest that they accumulated at least 10.0 and 3.7 m of reef framework during the Holocene, respectively^38,41,42^.

In shallow-water environments of the western Atlantic, including southern Florida, the elkhorn coral *Acropora palmata* was until recently the dominant reef-building coral during the late Quaternary^31,43,44^ (Fig. 1, Supplementary Fig. S3) and the presence of *A. palmata* reef framework is generally associated with periods of active reef accretion in the past^9,31,44–46^. We therefore used the ages of sub-fossil *A. palmata* to determine the timing and spatial extent of reef-building along the IR and OR of the SFCRT during the Holocene (Fig. 2). Data from the less-developed MR^38^ and from other coral species are included for comparison, but were not used to draw major conclusions about the history of reef development.

We collected and radiometrically dated 62 new samples, including 52 *A. palmata*, from several previously unsampled locations along the SFCRT: 16 from the reef-surface in central and northern Palm Beach County and 46 from the reef-surface and reef framework in southern Miami-Dade County. We combined these data with 64 previously published ages from reefs in Broward County^38,41,42,47,48^. We also included 15 ages from the deep-water ‘Outlier Reef’ off Fowey Rocks, in south Miami, which grew contemporaneously with the OR^49^ and is structurally analogous, even though it is considered to be part of the FKRT^38^. The full dataset of radiometric ages is provided in Toth et al.^50^ (https://coastal.er.usgs.gov/data-release/doi-P9Z21NMU/) and the data used in this study are summarized in Table S1. Although we describe the specific locations where samples were collected in the following sections, our results focus on summarizing the history of reef development on the IR and OR within three subregions of the SFCRT: Palm Beach County (“Palm Beach”), Broward County (“Broward”), and Miami-Dade County (“Miami”). The aggregated data form an extensive dataset of Holocene reef development across a latitudinal gradient spanning the full ~ 120 km of the SFCRT.

### Expansion and contraction of the SFCRT

Unlike the FKRT, which largely grew atop antecedent, late-Pleistocene reef framework^31,37,51^, there is no evidence of significant Pleistocene reef growth along the SFCRT. Indeed, the northern end of the FKRT is the limit of both contemporary reef development in southern Florida and of Late Pleistocene reefs^31^ (~ 400–125 ka). From Fowey Rocks north, a combination of coquina ridges and mixed carbonate/siliciclastic sandstones serve as the bedrock for the Holocene SFCRT^9,38,42,51^. The absence of Pleistocene reef deposits within the bedrock of the SFCRT suggests that reef growth was largely restricted to the lowest latitudes of southern Florida for much of the late Quaternary.

Beginning ~ 10 ka, however, the latitudinal range of reefs dominated by *A. palmata* expanded into shallow-water environments from south Miami to central Palm Beach County (Fig. 2; see Supplementary Discussion). Reef-accretion rates during the Early Holocene (~ 11.7–8.2 ka) ranged from 5.0 to 14.8 m per thousand years (m ky^−1^) and averaged 8.3 m ky^−1^ (± 3.8 standard error [SE]; Table 1), rivaling the most rapid rates of reef growth in the western Atlantic^12,45,46^. This pace of reef accretion would have been sufficient for the reefs to keep up^52^ with rapid Early Holocene sea-level rise^47,53^ (Supplementary Fig. S4), suggesting that environmental conditions in the nearshore habitats of southeast Florida were favorable for reef development at the time.

**Table 1:** Vertical accretion rates of *Acropora palmata*-dominated reefs on the Southeast Florida Continental Reef Tract.

Period	Subregion	Location	Sequence	Reef	Age range (ky BP)	Elevation range (m bMSL)	Accretion rate (m ky^−1^)
Early Holocene	Miami	Fowey Rocks	BP-FR-1	Outlier	10.4–9.1	14.0–22.3	6.3
	Miami	Fowey Rocks	BP-FR-2	Outlier	10.3–9.8	30–22.4	14.8
	Miami	Fowey Rocks	BP-FR-2	Outlier	9.8–9.4	22.4–19.4	8.0
	Miami	Fowey Rocks	BP-FR-2	Outlier	9.4–8.0	19.4–16.3	2.1*
	Broward	Pipeline Trench	BR-OR-PT-B	OR	10.8–8.8	26.5–16.5	5.0
	Broward	Pipeline Trench	BR-OR-PT-C	OR	10.1–9.4	24.5–18.0	7.4
Middle Holocene	Miami	Port Miami	PM-25 mE	OR	8.3–7.1	14.4–10.7	3.6
	Broward	Broward IR	BR-IR-B-1	IR	6.4–6.0	10.4–8.3	5.0
	Broward	Caves Reef	BR-IR-CR-9	IR	6.0–5.8	6.8–6.2	5.0
	Broward	Caves Reef	BR-IR-CR-10	IR	7.1–6.5	8.8–8.1	1.1
	Broward	Caves Reef	BR-IR-CR-10	IR	6.5–6.4	8.1–7.7	2.7
	Broward	Caves Reef	BR-IR-CR-10	IR	6.4–6.2	7.7–6.9	7.5
	Broward	Caves Reef	BR-IR-CR-15	IR	7.1–6.2	8.7–7.1	2.0
	Broward	Caves Reef	BR-IR-CR-15	IR	7.6–7.1	9.6–8.7	1.7
	Broward	Caves Reef	BR-IR-CR-16	IR	6.7–6.3	8.7–7.5	3.2

Accretion rates are reported in meters per thousand years (m ky^−1^) for dated reef sequences (OR = Outer Reef and IR = Inner Reef) during the Early and Middle Holocene (see “Methods” and Supplementary Discussion). Age ranges represent the lower and upper ages of each interval over which accretion rates were calculated. The ranges of elevations of samples used to calculate accretion are given in meters below modern mean sea level (m bMSL). The accretion rate indicated by an asterisk was not included in statistical analyses because the upper age of the sequence was not from *A. palmata* (see Supplementary Discussion).

**Figure 3:**
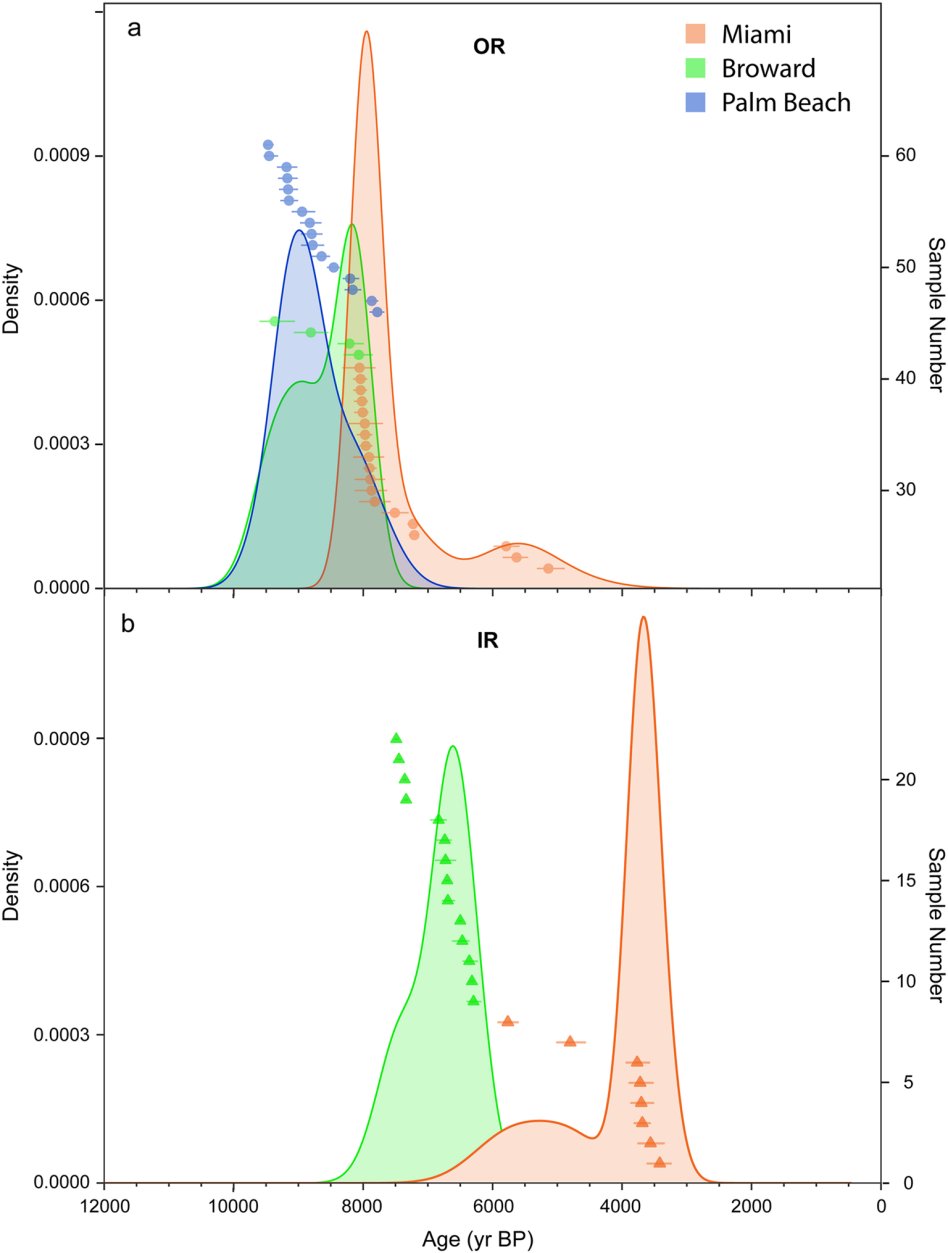
Kernel Density Estimations (KDEs) of when reef development terminated throughout the Southeast Florida Continental Reef Tract. The KDEs (shaded distributions) are estimates of the probability density functions of the distribution of *Acropora palmata* ages (points; horizontal uncertainties are ± 2σ) from within 1 m of the reef surface on (**a**) the Outer Reef (OR) and (**b**) the Inner Reef (IR). The starting bandwidth of the KDE analysis was set to 300 years based on the average total uncertainty of the ages (see “Methods”). The KDE plots were generated using the Isoplot package^88^ in RStudio^90^.

During the Middle to Late Holocene (8.2–4.2 ka and 4.2 ka–present, respectively) the SFCRT backstepped inshore and contracted to the south (Fig. 2). We used a non-parametric Kernel Density Estimate analysis (KDE; see “Methods”) to construct probability distributions of the surface ages from the IR and OR in each subregion. We interpret the peaks of those distributions to represent the last period of reef development at each location before reef accretion terminated. Reef accretion on the OR ceased throughout the SFCRT by the beginning of the Middle Holocene (Fig. 3a); however, the peak of the last period of reef growth on the OR in Miami, at 7.9 ka (95% confidence interval (CI) of KDE: 8.5–5.1 ka), occurred significantly later than in Broward (8.2 ka; 95% CI of KDE: 9.8–7.7 ka) or Palm Beach (9.0 ka; 95% CI of KDE: 9.8–7.5 ka; Kruskal–Wallis test: H_2_ = 18.47, p < 0.001; Nemenyi test: p < 0.001). The youngest ages for this final period of reef growth at each location were 7.2, 8.1, and 7.8 ka, respectively. There was a second peak of *A. palmata* ages on the OR in Miami at ~ 5.5 ka (± 0.2 SE; Fig. 3a), but it is not clear whether this represents a resumption of reef development or a short-lived, isolated population.

By the beginning of the Middle Holocene, rising sea level had flooded new shallow-water habitats throughout southeast Florida^53^, allowing the IR to initiate 1–2 km inshore of the OR in some locations^40,42^; however, the extent and duration of the inshore expansion of the SFCRT varied by latitude (Fig. 2). The most complete records of IR development, from central Broward, suggest that accretion initiated on the IR by ~ 8 ka^42^. The average accretion rate of *A. palmata* reefs there was ~ 3.5 m ky^−1^ (± 0.8 SE; Fig. 2; Table 1), which is comparable to average Holocene accretion rates elsewhere in the western Atlantic^12,45,46^.

We were only able to collect reef-surface samples from the Miami IR and cannot directly evaluate when *A. palmata* reefs established there; however, the similarity of the depth of the Pleistocene bedrock in this subregion (− 10 m MSL^51^) to the depths of the initiation surfaces on the IR of Broward (− 9 to − 12 m MSL)^38,42^, suggests that the IR likely established around the same time in both locations (see Supplementary Discussion). In contrast, the IR in Miami continued growing significantly longer—nearly 3 ky longer—after the Broward IR had shut down (Fig. 3b; youngest ages: 3.1 versus 5.7; peak of KDE: 3.2 versus 6.1, 95% CIs of KDE: 5.5–2.5 and 7.4–5.5 ka, respectively ; Mann–Whitney U test: U = 112, p < 0.001).

The IR is absent north of the Hillsboro Inlet in northern Broward County (Fig. 2; Supplementary Fig. S1)^40^, likely because of the lack of hardbottom antecedent substrata and abundance of unconsolidated sediments inshore of the OR at the northern limits of the SFCRT^38^. Indeed, a seismic profile off Delray Beach (26.5° N) in southern Palm Beach County interpreted by Finkl et al.^54^ suggested that the shallowest antecedent substrate available inshore of the OR there is at a depth of approximately 25 m. That would have been too deep for *A. palmata* reefs to establish during the Middle Holocene (i.e., ~ 13–20 m paleodepth from 8.2–6 ka)^53^. The only shallower inshore, consolidated substrate is the NRC, which is presently at a depth of around -4 m MSL^40^, but this feature would not have flooded until ~ 5 ka^40,53^.

### Sea-level change and reef development

**Figure 4:**
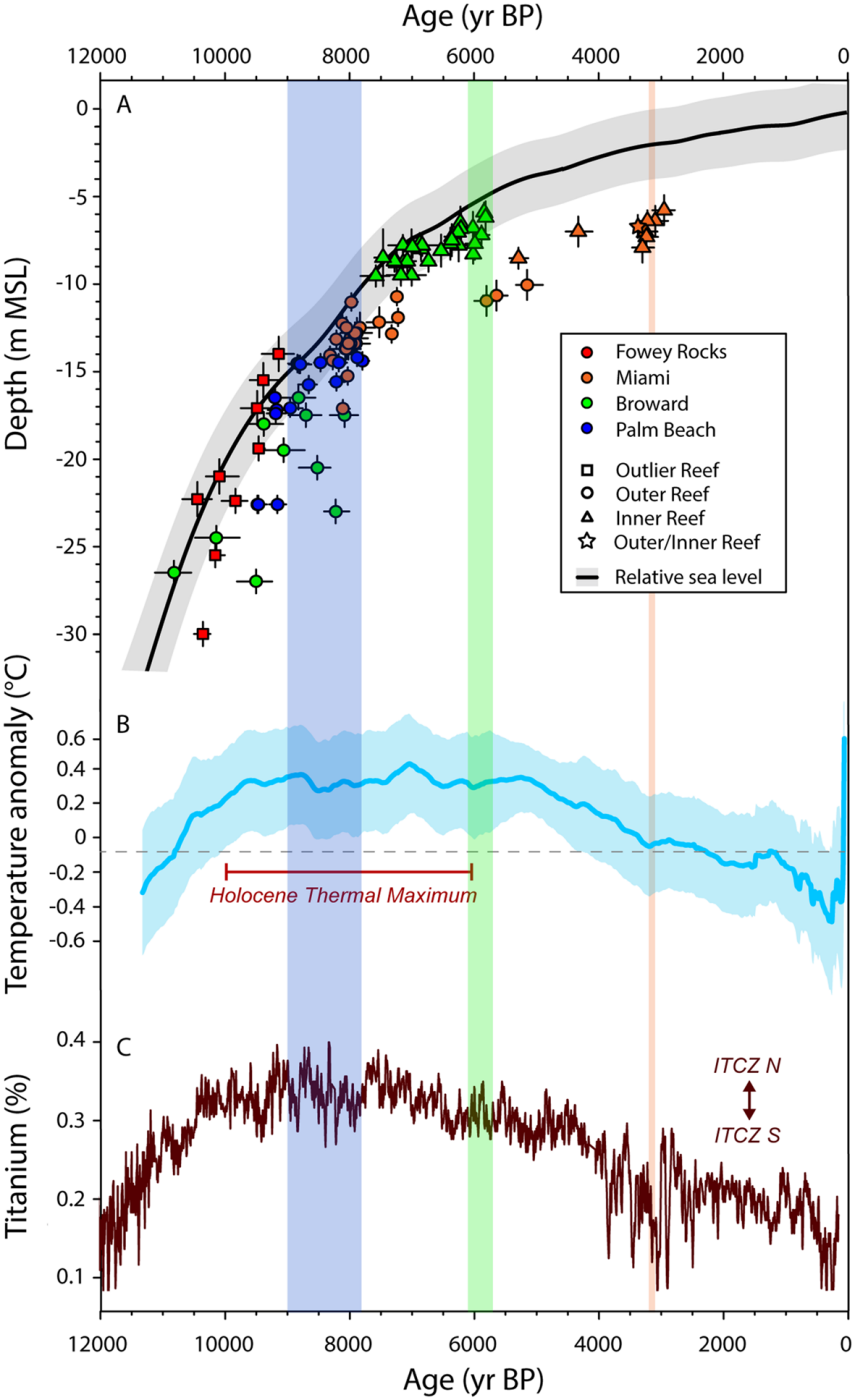
Reef growth by *Acropora palmata* on the Southeast Florida Continental Reef Tract compared with Holocene sea-level and climate variability. **(A)** *A. palmata* ages (± 95% confidence intervals [CIs], horizontal uncertainties) versus Holocene relative sea level in southern Florida^53^ plotted by depth relative to MSL (± 95% CI). Vertical uncertainties for *A. palmata* ages are 95% CIs of the root-sum-squares of estimated elevational uncertainties (see Toth et al.^50^). **(B)** Global composite of Holocene temperature anomalies^24^ (± 95% CI; see Supplementary Discussion). **(c)** Variability in the position of the inter-tropical convergence zone (ITCZ) inferred from Titanium flux to the Cariaco Basin (Ti%)^25^. Vertical shading represents the timing of reef termination in each subregion (width of shading is range from peak of KDE to youngest age at each location).

Before high-resolution data from the Broward IR were available, some researchers^55,56^ suggested that the > 500-yr gap and difference in elevation between published ages from the OR^41,47^ and IR^48^ of the SFCRT indicated that rapid, 6- to 7-m sea-level rise at ~ 8 ka shut down the development of the OR by reef-drowning. We revaluate this hypothesis in the context of our expanded dataset of *A. palmata* ages from southern Florida and recent sea-level reconstructions for the region^53,57^. The data we present on sea level in south Florida during the Holocene (Fig. 4A) are from a high-resolution (~ 50-y) output of the empirical statistical model of Holocene relative sea-level variability developed by Khan et al.^53^ (outputs published in Toth et al.^58^). Although we present the predictions of this model for the south Florida subregion, the model incorporates spatial correlations in sea level and, therefore, is reflective of regional trends from sea-level proxy data collected throughout the tropical western Atlantic^53^.

Several recent studies of Holocene sea level from the western Atlantic support the conclusion that sea level rose gradually over the transition from the Early to Middle Holocene^49,53,57^ (Fig. 4A), with modeled rates of sea-level rise of ~ 4.2 m ky^−1^ from ~ 8.5 to 7.5 ka in south Florida^53^. The empirical data are also consistent with the trends in sea-level rise predicted by regional geophysical models^57^. There is some evidence based on sea-level reconstructions from the northern Gulf of Mexico of an abrupt, centennial-scale rise in sea level associated with the draining of glacial Lake Agassiz during the 8.2 ka cooling event^60^. Although it is possible that this period of rapid sea-level rise may not have been detected in the existing sea-level models, the predicted magnitude of sea-level change in the tropical western Atlantic associated with that event would have been on the scale of decimeters rather than meters (i.e., only 20–40 cm in south Florida)^60^. Together, these reconstructions suggest that although high rates of sea-level rise at ~ 8.2 ka have been implicated as a driver of reef demise in a number of locations around the world^15,55,56^, the evidence for an abrupt sea-level jump at this time that would have been large enough to cause reef drowning is not robust in many locations^59^, including southern Florida^49^.

New data demonstrate that the early phases of IR growth in Broward were contemporaneous with the final period of reef growth on the OR^42^ (Fig. 2), suggesting that the gap in ages used to hypothesize a sea-level jump was a sampling artifact^49^. The elevations of the youngest *A. palmata* samples from the OR were ~ 4.5 and 2.2 m deeper than the oldest samples from the IR in Broward and Miami, respectively; however, the paleodepths of the OR would still have been within the 0 to ~ 5 m depth range preferred by the species^47^ in all three subregions (~ 4.1 m in Palm Beach at 7.8 ka , ~ 5.0 m in Broward at 8.1 ka, and ~ 2.7 m in Miami at 7.2 ka based on the Khan et al.^53^ sea-level reconstruction) and growth of the OR was keeping pace with sea level at the time of its shutdown (Table 1; Fig. 4A; Supplementary Fig. S3). There is also no evidence on the OR of a deepening-upward transition from *A. palmata* to massive corals that would have been characteristic of a reef-drowning event^52^. Instead, the upper surfaces of the OR were dominated by *A. palmata*, indicating their continued position near sea level^47^ (see “Methods”; Supplementary Fig. S3). Together, this evidence suggests that a driver other than sea-level change is needed to explain the termination of reef development on the OR of the SFCRT.

Similarly, although rising sea level promoted the initiation of the IR in some locations by flooding antecedent surfaces inshore of the OR, sea level cannot explain the demise of the IR. Accretion during the Middle Holocene on the IR was significantly slower than during the Early Holocene on the OR in Broward (Table 1; t-test: t_11_ = 2.96, p = 0.013), because of the relatively lower rates of sea-level rise after ~ 8 ka (3.9–2.1 m ky^−1^ from 8.1–5.8 ka)^53^; however, rates of *A. palmata* accretion on the IR continued to keep pace with sea level throughout the Middle Holocene (Supplementary Fig. S3). This, combined with the fact that many of the records from the Broward IR show a shallowing-upward transition from massive corals to *A. palmata*^42^, precludes the possibility of reef drowning^38^. The mean depth of the surface of the IR ranges between ~ 8 and 10 m at present^40^, suggesting that reef growth on the IR also has not been suppressed by a lack of accommodation space (cf. Toth et al.^9^).

Another hypothesis for the shutdown of Early Holocene reefs in the western Atlantic was that resuspension of terrigenous sediments from sea-level flooding of inshore areas caused reef drowning due to light limitation and eutrophication^41,52,61,62^. By the time the offshore reefs stopped growing, however, reef growth had initiated in inshore environments, where turbidity and nutrient loading would have been highest, which negates this hypothesis^42,46,62^ (Fig. 3). Although reef backstepping can certainly occur as a result of either rapid sea-level rise^15,55,56^ or an inimical offshore environment^52,61^, our data from the SFCRT support the conclusion that it can also occur in the absence of these drivers. In many cases, backstepping may simply be a part of the natural evolution of reef ecosystems as rising sea level creates new habitats inshore^46,62,63^. It is unclear what ultimately caused the OR in Broward and Miami to be abandoned when reef development continued on the IR in those locations, but similar scenarios of unexplained reef demise in other western Atlantic locations (e.g., the U.S. Virgin Islands and Puerto Rico) suggests a common, yet unidentified, driver^46,62^.

### Climate and the latitudinal shutdown of the SFCRT

Whereas *A. palmata* reef growth off Miami was continuous from ~ 10–3 ka, in Broward, reef development ceased by ~ 5.7 ka. At the northern limits of the SFCRT in Palm Beach, reefs only grew until ~ 7.8 ka (Figs. 2, 3). This produced a latitudinal gradient in the timing of reef shutdown (Supplementary Fig. S4; linear regression: F_1,36_ = 247.4, p < 0.001, r^2^ = 0.87, Termination Age = 5515.1*Latitude − 138024.5). Although the development of the SFCRT was controlled by the interaction between antecedent geomorphology, sea-level rise, and climate, we argue that climate was the primary driver of its contraction and eventual demise.

The global distribution of coral reefs is generally limited to locations where minimum seawater temperatures exceed ~ 18 °C ^11^. In southern Florida, temperatures typically remain above this minimum, but winter cold-fronts periodically push reefs below that threshold^31^, limiting modern reef development in the region^29^. For example in January of 1977 and 2010, Florida’s reefs experienced two of the most extreme cold events on record, with prolonged low-temperature excursions causing significant coral mortality throughout much of the region^32,34,35^. Because *A. palmata* is particularly cold-sensitive, even more moderate cold fronts would have been sufficient to limit its historic distribution to habitats south of the SFCRT^31,33^ and suppress significant reef accretion over millennial timescales^29^. Currently, extreme cold fronts impact southern Florida with a period of ~ 20 years^18^; however, it is likely that climate modulated the periodicity and geography of cold-front impacts during the Holocene.

**Figure 5:**
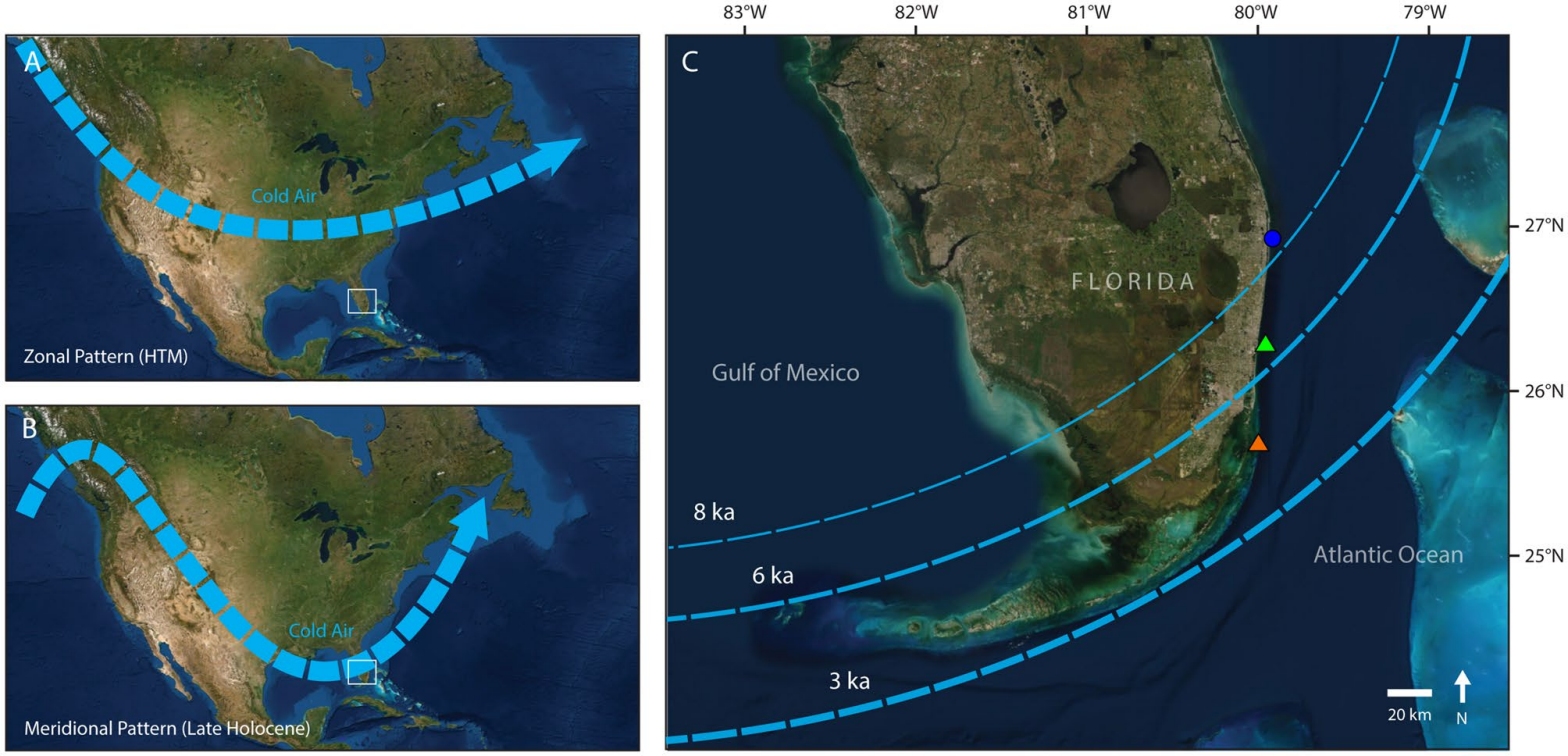
Diagram of the two dominant patterns of winter atmospheric circulation over North America in relation to our study area: **(a)** dominance of zonal flow, which suppresses the transport of cold air to the southeastern United States and **(b)** dominance of meridional flow, which is associated with increased frequency of winter cold fronts reaching the southern United States including to southern Florida (bounding box)^26^. Southern Florida is expanded in **(c)**, which shows the approximate trajectory (~ 45° angle) of winter cold fronts in this region^91^ in relation to our sampling locations on the Palm Beach Outer Reef (blue circle), the Broward Inner Reef (green triangle), and the Miami Inner Reef (orange triangle). This panel provides a hypothetical conceptual model (dashed lines) of when extreme winter cold fronts would reach different latitudes in southeast Florida with high enough frequency to suppress reef development. We suggest that extreme winter weather would have impacted increasingly southern latitudes over the Holocene in response to climate forcing. Cold front intensity may have also increased over time, a trend represented by the thickness of the dashed lines. Map image is the intellectual property of Esri and is used herein under license. Copyright 2020 Esri and its licensors. All rights reserved.

The primary control on the frequency of winter cold fronts reaching southern Florida is the intensity of meridional versus zonal atmospheric circulation over North America (Fig. 5), with strong meridional flow forcing increased penetration of cold fronts to the south^18,26^ (see Supplementary Discussion). Because these subregional patterns of atmospheric circulation are produced by broader-scale drivers of regional climate^27^, changes in meridional flow are reflected in various paleoclimate records^25,27,64^. For example, a more southerly position of the inter-tropical convergence zone (ITCZ) is associated with increased meridional circulation and a southerly shift of the polar jet stream^25,27^. Conversely, a more northerly position of the ITCZ is associated with increased zonal circulation and a northerly shift of the polar jet stream^25,27^. High-resolution reconstructions of local, millennial-scale temperature variability from the marine environments of southeast Florida are not available at present and we were unable to directly reconstruct climate variability in this study due to limited sampling of the western Atlantic corals that have been shown to produce high-fidelity paleoclimate data: *Orbicella faveolata* and *Siderastrea siderea*^28^. For these reasons, and because our *A. palmata* ages only record the timing of reef shutdown rather than the full history of reef development in many locations, we were not able to statistically evaluate the relationship between climate and reef-building. Instead, for the following discussion we rely on broad correlations between reef development and Holocene variability in global-scale temperature^23,24^ (Fig. 4B), the mean position of the ITCZ^25^ (Fig. 4C), and other paleorecords indicative of changes in meridional circulation over the eastern United States^64,65^ (see Supplementary Discussion) to evaluate the likely role of cold-front variability on the latitudinal contraction of reef-building in south Florida. We acknowledge that there is some debate about the timing and spatial fingerprint of temperature changes related to the HTM; however, the general trend of Early to Middle Holocene warming followed by Late Holocene cooling suggested by the composite record we rely on here^24^ (Fig. 4B), was reproduced by an updated, more comprehensive (but lower-resolution) reconstruction^66^ (Supplementary Fig. S5), validating the occurrence of a global HTM. Furthermore, recent studies have demonstrated that oceanographic and climatic variability in the subtropical habitats of south Florida are strongly linked to broader-scale changes in Northern-Atlantic climate^28,67^ (see Supplementary Discussion), which is the primary driver of the global signature of the HTM in those records. These lines of evidence suggest that the global trends in Holocene temperature reflected in Fig. 4B provide a reasonable proxy for mean temperature variability in south Florida.

The expansion of the Florida Reef Tract throughout southeast Florida during the Early Holocene coincided with the onset of warmer global temperatures associated with the HTM beginning ~ 10 ka^23,24,29^ (Fig. 4B; Supplementary Fig. S5). The ITCZ also occupied its northernmost position of the Holocene during this interval^25^ (Fig. 4C), which would have constrained the polar jet stream to high latitudes^27^. Furthermore, records of marine aerosol flux to the Greenland ice sheet indicate that by ~ 10 ka meridional circulation was weak^64^ (see Supplementary Discussion). All this suggests that the HTM was likely a time of warmer average temperatures and fewer cold fronts in southeast Florida. We hypothesize that this more tropical climate allowed for the northern expansion of *A. palmata* reef development throughout the SFCRT.

The termination of reef development at the northernmost limit of the SFCRT in Palm Beach between ~ 9.0 and 7.8 ka (Figs. 2, 3; peak of KDE and youngest age; see “Methods”), coincides with an interval of enhanced meridional circulation inferred from the Greenland record (8.8–7.8 ka)^64^, a brief southerly excursion of the ITCZ^25^ (Fig. 4C), and moderate cooling of the North Atlantic^23^ (the 8.2-ka event; Fig. 4B). Although the occurrence and magnitude of lower-latitude cooling associated with the 8.2-ka event is debatable (Supplementary Fig. S5), the changes in atmospheric circulation at this time would likely have produced cooler winters at the northernmost extent of the SFCRT (Fig. 5c). That winter cooling, we argue, extirpated cold-sensitive *A. palmata* populations^18,29^. By the time conditions once again became favorable for *A. palmata* to recolonize the Palm Beach OR, sea levels would have risen by nearly 5 m (from 9.0 to 7.8 ka; Fig. 4A)^53^, pushing those habitats beyond the preferred depth range of the species^47^. With no suitable shallower inshore habitats available in this subregion^38^, *Acropora*-dominated reefs would have been unable to reestablish after 7.8 ka (see Supplementary Discussion).

Reef growth continued further south on the IR of Broward until 6.1–5.7 ka (Figs. 2, 3), when a second major period of enhanced meridional circulation was recorded in Greenland^64^ (6.1–5.0 ka) and the ITCZ had continued to move south^25^ (Fig. 4C). Whereas the record from Greenland is suggestive of a short-term increase in the frequency and/or intensity of cold-fronts reaching south Florida, the longer-term shift in the position of the ITCZ may suggest that the frequency at which cold fronts were reaching lower latitudes was also gradually increasing over time. Thus, whereas Broward’s reefs were not critically affected by the period of colder winters that suppressed reef development in Palm Beach between ~ 9.0 and 7.8 ka, we hypothesize that by 6.1–5.7 ka severe cold fronts were reaching the lower-latitude reefs in Broward with high enough frequency to impair reef development (Fig. 5c). By this time, the global climate had also begun to cool following the peak of the HTM^24^ (Fig. 4B; Supplementary Fig. S5). A cooler mean climate in southeast Florida by the Middle Holocene may have brought average temperatures in Broward closer to the lower thermal threshold for reef development, making them more vulnerable to the later increase in cold-front frequency, leading to their shutdown by 5.7 ka.

Global temperatures cooled substantially during the Late Holocene^24^ (Fig. 4B; but see Supplementary Fig. S5), which would have likely brought reefs at the lowest latitudes of southeast Florida near their lower thermal threshold. The beginning of the Late Holocene was also characterized by a phase shift in atmospheric circulation from zonal to meridional dominance^27^, suggesting cold-front frequency in the lowest latitudes of southern Florida would have increased substantially relative to the Middle Holocene^26^ (Fig. 5c). This inference is supported by another interval of enhanced marine-aerosol flux in Greenland^64^ (3.1–2.4 ka), a negative North Atlantic Oscillation^18,26,65^ (4.5–2.0 ka), and extreme southern excursions and high variability in the position of the ITCZ^25^ (Fig. 4C). We hypothesize that these changes were responsible for the termination of reef building on the southernmost reefs of the SFCRT by 3.2–3.1 ka (Figs. 2, 3). The demise of reefs on the SFCRT around 3.0 ka also coincides with the shutdown of reef building on the FKRT further south^9^, suggesting that climate has been suppressing reef growth throughout southern Florida for at least the last three millennia.

Although reef communities established on the less-developed MR habitats^38^ and inshore of the IR on the NRC^50^ during the Late Holocene, there is no evidence of significant reef accretion at these locations (Supplementary Table S2; Supplementary Fig. S3). Whereas lower accommodation space as a result of minimal Late Holocene sea-level rise^53^ may have contributed to the lack of vertical reef accretion on the NRC, populations of reef-building corals were also extirpated from those reefs by 2 ka, which was is also likely a result of cooling. The depth of the MR is intermediate to that of the IR and OR and the average elevation of the MR is between -14.1 and -16.9 m MSL at present^40^, suggesting that accommodation space would not have been limiting there. Based on the limited data available, there is no evidence that *A. palmata* reefs ever formed on the MR, and the rates of reef accretion by massive corals there during the Middle to Late Holocene were significantly lower than on the IR (t-test: t_6_ = 3.34, p = 0.008). The two Late-Holocene ages we obtained from the MR in Miami (1.4 and 0.5 ka^50^) were from unattached *Mancinia areolata* colonies that are commonly found in sea grass habitats, suggesting that the MR may represent a back-reef habitat that formed when the OR was accreting, rather than a well-developed fore-reef like the IR and OR.

Most modern reefs throughout southern Florida are dominated by generalist or weedy corals^44^, primarily *Siderastrea siderea, Porites astreoides, Millepora alcicornis, Stephanocoenia intersepta,* and *Montastraea cavernosa* in southeast Florida^68^; however, most of these species have relatively low capacity for carbonate production and they have not contributed significantly to the construction of Florida’s reefs^44^. Furthermore, in southeast Florida, recent cold-stress events were associated with increases in the abundance of non-calcifying organisms including gorgonians and fleshy macroalgae^32^. It is likely that the same suite of taxa would have colonized the relict reefs of the SFCRT during the Holocene following cold-related mortality of *A. palmata*, but unlike *A. palmata*, these non-reef-building taxa would have made little if any subsequent contribution to the reef framework.

### Lessons from marginal reef environments

Most reefs in the tropical western Atlantic grew more-or-less continuously during the Holocene, and reef development in many parts of the world was more strongly influenced by sea level than by climate^45,46,59,69^. In the marginal, high-latitude environments of southeast Florida, sea-level rise facilitated backstepping of reefs into inshore habitats during the Middle Holocene, but latitudinal expansion and contraction of Florida’s reefs appears to have been most strongly modulated by climatic variability^9,29^. The relatively warm, stable climate of the HTM allowed reefs in Florida and other high-latitude locations^15–17,29,30^ to expand their ranges poleward during the Early Holocene. As the climate cooled and the frequency of winter cold fronts increased, however, the SFCRT contracted equatorward. The shutdown of reef-building at the southernmost limits of the SFCRT at ~ 3 ka coincides with the termination of reef-building throughout the FKRT, which also appears to have occurred as a result of cooling^9^. A recent reconstruction of reef development from subtropical reefs in southeastern China^16^ likewise implicated cooling following the HTM as a likely cause of declines in development, which, in combination with the records from southern Florida, suggests that subtropical reefs may generally be more sensitive to climatic variability than those in more tropical locations. In addition, marginal reefs in the eastern tropical Pacific that experienced strong, seasonal, cold-water upwelling were more vulnerable to millennial-scale climatic perturbations during the Holocene than reefs with more stable annual temperatures (i.e., in the Gulf of Panama^8^ and the Gulf of Papagayo, Costa Rica^13^). Whereas moderate levels of thermal variability can increase the resilience of reefs to modern climatic extremes^21,22,70,71^, these studies of Holocene reef development suggest that in marginal habitats, where variability in the physical environment is especially high, periodic disturbances may be more likely to push reefs past critical environmental thresholds for continued survival and reef-building^9,11,22,71^.

Although populations of thermally sensitive acroporid corals have periodically expanded their ranges north to the SFCRT in recent decades in response to contemporary warming^29^, and historic records suggest that many reefs on the FKRT had high coral cover as recently as the early 1970s^31^, carbonate production and reef accretion have remained negligible in most locations throughout southern Florida^9,36^. Furthermore, recent thermal- stress events and the devastating outbreak of stony coral tissue loss disease have significantly reduced the abundance of reef-building corals throughout the SFCRT^32,72^. As a result, the reef framework constructed over millennia is now rapidly eroding^9,73,74^. Active management and the restoration of reef-building corals has the potential to re-establish some key ecological processes and mitigate the problem of erosion on Florida’s reefs^43,44^, but a resumption of reef-building on the SFCRT is unlikely, at least on decadal to centennial scales. Whereas coral populations have the potential to respond to favorable conditions in the short-term, the geological process of reef-building is more vulnerable to environmental variability and more difficult to restore once it is lost^9^.

Although the return to a warmer mean climate could be favorable for high-latitude reef development^7,15–17,19,29,30^, analogizing future climate to the HTM may be a false comparison. Anthropogenic climate change is not simply driving global warming: it is amplifying changes in the frequency, intensity, and geography of extreme-weather events^19,75,76^. For example, the increasing frequency and severity of high-temperature extremes will continue to cause widespread coral bleaching and outbreaks of infectious coral disease around the world^1,2,69,72,76^, and even putative cooler-water coral refugia such as high-latitude reefs, mesophotic reefs, and upwelling zones are not immune to these impacts^20^. Because the coral assemblages in these marginal environments are already depauperate and are isolated from the diminishing source populations elsewhere in the Caribbean, they may be even less resilient to climate change and other anthropogenic impacts than most ‘mainstream’ reefs^20^. Additionally, as a result of the disproportionate contemporary warming of the high-latitude northern Hemisphere (i.e., Arctic Amplification), climate change may be amplifying the trend of suppressed zonal circulation^77,78^, favoring meridional flow patterns that are reminiscent of the longer-term shift that occurred during the Middle to Late Holocene^27,77,78^ (Fig. 5). These changes have produced a weaker and more “wobbly” jet stream and have been linked to increases in the frequency of extreme winter cold events in the mid-latitudes and subtropics since the 1990s^77,78^. Many models suggest that climate change will continue to increase the frequency of winter extremes in a number of locations, including the southeastern United States in the future^75^. The impacts of increased thermal variability—in both directions—may, therefore, be most keenly felt in subtropical environments like southeast Florida^19^, although more work is needed to assess the generality of this pattern. We conclude that climate change will likely be more limiting to framework-building corals in subtropical environments than in tropical environments, negating the potential for subtropical habitats to support renewed reef development.

## Methods

### Sample descriptions

#### Samples from previous studies

The earliest data from the SFCRT reef subsurface were provided by Lighty et al.^41,47^ from a 450-m-long trench excavated through the OR for construction of a wastewater pipeline off northern Broward County. Five distinct facies were recognized within the internal structure of the OR, clearly demonstrating that it possessed a classical Caribbean-reef zonation dominated at the reef-crest by *A. palmata*. Ten *A. palmata* samples of Early Holocene age were collected from − 16.5 to − 27.0 m MSL^41,47^. Similarly, Shinn et al.^51^ described massive-coral facies of Middle Holocene age from offshore northern Miami-Dade County in outcrop from a dredge excavation through an “intermediate ridge” at − 13.7 m MSL (i.e., between the MR and OR, sensu Banks et al.^38^) and from a wastewater-pipe excavation through the IR at − 9.8 m MSL (samples MD-IR-BH-16.1 and MD-IR-ST-9.8, respectively in Toth et al.^50^).

A series of more recent studies from reefs off central and southern Broward County^38,42^ detailed the internal composition of the IR. A trench caused by the grounding of the submarine *USS Memphis*^79,80^ afforded yet another opportunity to observe and date intact reef framework (Supplementary Fig. S2c). Researchers reported that the IR was at least 3 m thick at this location and consisted of mixed *A. palmata* and massive-coral framework^42^. Ten samples collected from the trench created by the grounding of the *USS Memphis* at − 7.8 to − 9.5 m MSL^48,80^, and an additional three samples from the same location dated by Stathakopoulos and Riegl^42^, were all of Middle Holocene age. Follow-up studies from several nearby locations on the IR using core-drilling techniques yielded similar results^38,42^.

#### Collection of reef-surface samples

Between August and November 2013, ABM and AEO collected a total of 12 reef-surface samples from four randomly selected sites on the OR offshore of Boynton Beach in Palm Beach County (26.51° N, 80.03°W). At each site, exposures of internal reef framework were haphazardly sampled within two randomly selected 10 × 10 m quadrats using a hammer and chisel. Eight representative samples from the site were selected for radiometric dating in this study. On 7 July 2019, ABM and AEO collected eight additional *A. palmata* samples from the upper surface of the OR offshore of West Palm Beach, Florida, ~ 20 km north of the Boynton Beach location. Three samples were collected from a dome-shaped reef with ~ 4.5 of m vertical relief called Turtle Rocks (26.72° N, 80.03°W), which marks the northernmost extent of the OR in Palm Beach County. Five samples were collected just south of that location from a 2-m-deep trench in the reef surface that had previously been dredged for a sewage outfall (26.70° N, 80.02°W; Supplementary Fig. S2d). The samples were collected using an underwater Nemo handheld drill with a 4-cm-diameter diamond-tipped drill bit. The water depth of each sample was recorded in the field using a digital dive computer and later tide-corrected to MSL using data obtained from the NOAA tide station at the Lake Worth Pier in Lake Worth, Florida (Station ID: 8722670; https://tidesandcurrents.noaa.gov/).

During the Fall of 2013, 17 permanent monitoring sites were established on the IR and OR off Miami Beach, Florida (25.75° N, 80.11°W) as part of the compliance monitoring for the Port Miami (Government Cut) Deepening Project^81^. There were nine stations on the IR and eight on the OR. At each site, three permanent transects were established by installing markers at 0, 10, and 20 m along each transect. In addition, three sediment stations were established adjacent to each transect at each site. To install permanent markers at each of the monitoring stations, a total of 12 holes were drilled into the reef surface with a hydraulic-powered rotary drill using a 2.5-cm-diameter coring bit (Supplementary Fig. S6). JHH and WFP collected a total of 204 core plugs that penetrated 5–10 cm into the reef surface. Of these, 96 cores were retrieved from the IR and 108 from the OR. Of the collected cores, 78 (72%) from the IR and 90 (94%) from the OR were composed of *A. palmata*. We haphazardly chose 10 cores from the IR and 16 cores from the OR for dating. WFP collected four additional surface samples by hand from other locations in south Miami: one *A. palmata* sample from the southern end of the IR and OR where the two reef-lines merge just south of Key Biscayne, but north of Fowey Rocks (25.64° N, 80.10°W), one *A. palmata* from the OR in the same area, and two *Ma. areolata* samples from the MR near Government Cut (25.75° N, 80.11°W).

We note that although we cannot definitively demonstrate that all of the surface samples of *A. palmata* we collected in this study were in their original growth positions, they were all collected from well-cemented reef framework, suggesting that they are likely in situ and not storm-ridge deposits. Furthermore, our observations of the exposed IR and OR in Miami (Government Cut; Fig. 1a) indicate that these reefs are primarily composed of in situ reef framework. Similarly, we cannot verify that the ages we used from previous studies were all from in situ corals^49,82^; however, those studies described sampling well-developed reef framework^38,41,42,47^ and the dated sequences generally had well-ordered chronologies^49,50^, suggesting that those samples were not allochthonous deposits.

#### Collection of reef-framework samples

On 8 May 2017, WFP, LTT, and MLR collected a total of 29 subfossil coral fragments by hand from the southern channel-wall of Government Cut, where the channel cuts through the in situ reef framework of Miami’s OR (Fig. 1a; Supplementary Fig. S2a,b; 25.76° N, − 80.09° W). Prior to sampling, divers laid out 50-m transects running east and west from a central point along the top surface of the channel wall. Sampling was conducted at the central point (0 m) and every 25 m along the transect lines. At each 25-m mark, divers haphazardly collected one or more coral samples near the top (− 12 m relative to MSL), middle (− 14 m MSL), and bottom (− 16 m MSL) of the channel wall. The water depth of each sample, as determined by dive computers, was recorded in the field. The in situ depth measurements were later tide-adjusted to depths relative to MSL using data from the NOAA tide station on Virginia Key, FL (Station ID: 8723214; https://tidesandcurrents.noaa.gov/), located 8 km SW of the site. For this study, 11 of the best-preserved samples of *A. palmata* and five samples of other coral species, representing a depth range of − 10.7 to − 17.1 m MSL, were selected for radiometric dating.

### Radiometric dating

We determined the ages of 62 of the newly collected sub-fossil corals from throughout southeast Florida: eight from the surface of the OR off West Palm Beach, eight surface samples from the OR off Boynton Beach, four surface corals collected from south Miami, 26 of the surface cores collected from the IR and OR around Government Cut, and 16 of the corals collected from within the OR framework of Government Cut^50^.

Samples free from any visually observable diagenetic alteration were collected from the internal skeletons of those corals using tile saws dedicated to that purpose at the USGS Saint Petersburg Coastal Marine Science Center or at Florida Atlantic University. All samples were sonicated in a bath of warm (~ 25–35 °C), deionized water for 15 min to remove detrital material from the skeletal matrix and were acid-etched to remove surficial contaminants prior to radiocarbon analysis. The samples from Boynton Beach were also soaked in a diluted 8% sodium hypochlorite solution to remove organics and pre-screened for evidence of diagenetic alteration using a combination of X-ray diffraction and petrographic analysis of thin sections. Those samples contained no detectable calcite and only minimal secondary aragonite.

All of the surface samples from West Palm Beach, eight of the reef-surface samples collected around Government Cut, and two of the Boynton Beach samples were dated by accelerator mass spectrometry (AMS) at the Woods Hole Oceanographic Institution’s National Ocean Sciences AMS Center (NOSAMS). The remaining six corals from Boynton Beach were dated by AMS at the Center for Applied Isotope Studies at the University of Georgia. The samples from south Miami were processed at the USGS Radiocarbon Laboratory in Reston, Virginia and were AMS-dated at the Lawrence Livermore National Laboratory. The remaining surface samples from around Government Cut were AMS dated at the Keck Carbon Cycle AMS Laboratory at UC Irvine using the rapid dating methodology described by Bush et al.^83^. Although this method results in higher analytical uncertainties (i.e., lower precision) than for samples dated using the standard AMS procedure, it has been shown to produce ages with high accuracy^84^. Dating methods for the ages derived in previous studies^38,41,42,47,48,51,85^ can be found in those publications (see also Toth et al.^58^ and Stathakopoulos and Toth^82^). Conventional radiocarbon ages were corrected for the fractionation of δ^13^C based on measured δ^13^C, or δ^13^C = 0 ± 4‰ if δ^13^C was not measured. All radiocarbon ages were calibrated to years before present (where ‘present’ is 1950 C.E.) using the Marine13 calibration curve^86^ in Calib 7.0.2 software (http://www.calib.org). In order to account for the temporally variable, local radiocarbon reservoir-age offset (ΔR) in southern Florida^67^, each radiocarbon age was assigned a predicted ΔR (see Toth et al.^50^) from an empirical model of Holocene ΔR variability developed by Toth et al.^67,87^. Two U-series ages from Stathakopoulos and Riegl^42^ were excluded from our analysis because elevated ^232^Th indicated the possibility of contamination by detrital thorium, and an additional sample from that dataset was excluded because it was likely transported from its original depositional environment^49,82^. All radiometric age data, sample depths (and uncertainties), and sample metadata are provided in Toth et al.^50^ (10.5066/P9Z21NMU).

### Determining reef-accretion rates and termination

Where possible, we used the ages from vertical sequences to estimate vertical accretion rates during intervals in the history of the SFCRT (see Supplementary Discussion). Accretion rates were calculated by dividing the depth interval over which a sequence was deposited by its timespan (lower minus upper age of that interval). Accretion rates from the OR of Miami were derived from a vertical transect of samples from *A. palmata* framework collected from Government Cut. For the previously published records^38,42,85^, we used published core logs^42,58^ to identify which intervals in the cores were dominated by *A. palmata*, massive corals, or mixed assemblages. Although we did not find a significant difference in accretion by massive, *A. palmata*, or mixed facies (Supplementary Discussion and summarized in Supplementary Table S2), for consistency, we focus our discussion on trends in accretion of *A. palmata* facies as this was the only taxon present throughout the entire Holocene record.

The time at which reef development terminated at each site was determined based on the ages of *A. palmata* samples collected within 1 m of the reef surface^50^. The timing of termination of the OR and IR in each subregion was visualized using non-parametric Kernel Density Estimation (KDE) using the IsoplotR package v.3.3^88^. KDE creates a smoothed probability density curve based on the weighted distances of data within a sliding probability distribution function (kernel) window. The shape of the kernel (width of the window) is defined by a bandwidth parameter within the KDE. The IsoplotR package uses an adaptive bandwidth modifier^89^ that increases the bandwidth where data are sparse and decreases the bandwidth where the data are dense^88^. In our KDE analysis, we used a starting bandwidth of 300 years, based on the mean total (positive plus negative) 2σ uncertainty in the radiometric ages of 287 years in the dataset. The peaks in the KDE represent clustering in the distribution of *A. palmata* ages near the reef surface, which we assume to represent the last period of reef development in any given location. Termination of reef accretion most likely would have occurred sometime between this peak and the youngest *A. palmata* age at that location. We used this range of ages as a conservative estimate of the timing of reef shutdown rather than simply relying on the youngest *A. palmata* ages at each location because minimum ages are subject to sampling biases.

### Statistical analyses

OR-termination ages were compared among subregions using a Kruskal–Wallis test and Nemenyi post-hoc test because the data were not normally distributed even after natural-log or square-root transformation. For this analysis, we did not include three *A. palmata* ages from the OR of Miami that were significantly younger (by > 2.3 ky on average) than the rest of the ages in the dataset (Mann–Whitney U test: U = 48, p = 0.002). These ages represent a separate, later interval when *A. palmata* was present on the Miami OR that would have biased the comparison among subregions. Without additional sampling it is not possible to determine if these ages represent the resumption of reef development on the Miami OR or a short-lived, isolated population. The timing of IR-termination in Broward and Miami was compared using a Mann–Whitney U test because the data were not normally distributed even after natural-log and square-root transformation. Final termination ages (the OR in Palm Beach and the IR in, separately, Broward and Miami) were compared along a latitudinal gradient using a linear regression. The residuals of the model were normally distributed (Shapiro–Wilk test: W = 0.95, p = 0.83) with untransformed data. Accretion rates of *A. palmata* reefs were compared between the Early and Middle Holocene in the Broward subregion using an independent t-test. The data were normal (Shapiro–Wilk test: W = 0.96, p = 0.82) and homogeneous (Levene’s test: F_1,11_ = 1.86, p = 0.20) after natural-log transformations. We also compared the rates of reef accretion during the Late Holocene by massive corals on the IR to the single estimate of reef accretion on the MR, 0.8 m ky^−1^ (Supplementary Table S2), using a one-sample t-test. The IR data were normal without transformation (Shapiro–Wilk test: W = 0.85, p = 0.12). All statistical analyses were conducted using RStudio v.3.6.3^90^.

## Supplementary Information

Supplementary Information.

## Data Availability

All data used in this study are published in USGS Data Releases (https://doi.org/10.5066/F7NV9HJX and 10.5066/P9Z21NMU) with FGDC-compliant metadata.
