# Supplementary material for: Climate and the latitudinal limits of subtropical reef development
Source: Sci Rep. 2021 Jun 22;11:13044. doi: 10.1038/s41598-021-87883-8 (PMC8219804; doi:10.1038/s41598-021-87883-8)
Supplement: Supplementary file 1 — Supplementary Information. [file 41598_2021_87883_MOESM1_ESM.pdf]

Scientific Reports

Supplementary Information for

Climate and the latitudinal limits of subtropical reef development

Lauren T. Toth[1]*, William F. Precht[2], Alexander B. Modys[3], Anastasios Stathakopoulos[1], Martha L. Robbart[2,4], J. Harold Hudson[5], Anton E. Oleinik[3], Bernhard M. Riegl[6], Eugene A. Shinn[7], Richard B. Aronson[8]

1. *U.S. Geological Survey St. Petersburg Coastal and Marine Science Center, St. Petersburg, FL*
2. *Marine and Coastal Programs, Dial Cordy & Associates, Inc., Miami, FL*
3. *Department of Geosciences, Florida Atlantic University, Boca Raton, FL*
4. *Independent Consultant, Glenmont, NY*
5. *Reef Tech, Miami, FL*
6. *Department of Marine and Environmental Sciences, Nova Southeastern University, Dania Beach, FL*
7. *University of South Florida, College of Marine Science, St. Petersburg, FL 33701*
8. *Department of Ocean Engineering and Marine Sciences, Florida Institute of Technology, Melbourne, FL*

*Corresponding author: ltoth@usgs.gov

Supplementary Information included in this file:

Supplementary Discussion

Supplementary Tables S1 and S2

Supplementary Figures S1–S6

Supplementary References

SUPPLEMENTARY DISCUSSION

Initiation of reef development in southeast Florida

Complete records of Holocene reef development are now available for the Broward Outer Reef (OR)[1,2] and the Broward Inner Reef (IR)[3,4], but we were only able to collect surface samples from the Palm Beach OR and the Miami IR in this study. Furthermore, the location where we sampled the Miami OR may not represent the full reef history there. As a result, we were unable to directly measure when reef growth began in these locations; however, we can infer the likely timing of reef initiation by comparing their geomorphology to analogous features elsewhere on the Southeast Florida Continental Reef Tract (SFCRT).

Although we were only able to collect surface samples from the OR in Palm Beach, those samples were collected across a depth range of 8.4 m (-22.6 to -14.2 m relative to mean sea level [MSL])[5], which is only slightly less than the reported 10-m thickness of the Broward OR[2]. Our ages from the Palm Beach OR range from 9.5–7.7 ka[5], which is within 700 years of the oldest age for the Broward OR from a sample likely to be *in situ* (from Sequence C of Lighty *et al.*[2])[6]. Overall, the similarity in the age and depth distributions of our samples from the Palm Beach OR to the Broward OR suggests that the timing of reef initiation was similar at the two locations: ~10 ka.

The oldest ages from the location where we sampled the OR in Miami, sampled just above the carbonate bedrock at -16.2 m MSL, were ~8.3 ka[5]; however, Lidar bathymetry suggests that the OR actually extends ~400 m seaward from this location into deeper water[7]. The deeper, outlier reef, located ~18 km south of the location where we sampled the Miami OR, initiated at 10.4 ka at a similar depth as the Broward OR (-22.3 to -30 m MSL versus -24.5 to -27 m MSL, respectively)[5]. It is likely, therefore, that our data from south Miami represent the terminal phase of reef growth

on the Miami OR and that the deeper portions of the OR, which we were not able to sample in this study, began growing during the Early Holocene, around 10 ka.

As with the OR samples from Palm Beach, we were only able to collect surface samples from the Miami IR. The oldest age of the samples from Miami is 5.3 ka and most cluster around 3.3 ka. Unlike the samples from Palm Beach, however, the samples from the Miami IR were collected over a narrow depth range of 2.7 m (-8.5 to -5.8 m MSL)[5]. We do not, therefore, have data from the majority of the lifespan of the Miami IR, which is why there is a gap in the record of *Acropora palmata* reef development in Miami from 7.2 to 3.3 ka (with the exception of the three samples from 5.3–4.5 ka); however, a colony of *Montastraea cavernosa* found just above the Pleistocene surface in a section of the south Miami IR exposed by a pipeline installation provides an estimate of the depth of the IR initiation surface in this region: approximately -10 m MSL[8]. As we discuss in the main text, this is similar to the depths-to-Pleistocene on the IR of Broward (-9 to -12 m MSL)[3,4], which suggests that both reefs would have likely initiated by the beginning of the Middle Holocene. Furthermore, surveys of the Miami IR within the Port Miami dredge exposure by WFP using underwater depth gauges suggested that the Holocene reef there extended to at least -15.2 m MSL (the depth of the channel), so the IR-initiation surface in some parts of Miami may be even deeper than in Broward.

Estimates of accretion rates

We estimated the rates of reef accretion during the Early Holocene from *A. palmata*-dominated reef framework at the Fowey Rocks Outlier Reef in south Miami[9,10] and from the two most reliable sequences sampled on the Broward OR (from Sequences B and C of Lighty *et al.*[2])[6]. All of the samples we collected from the reefs in Palm Beach were surface samples, so it was not possible to derive estimates of reef accretion for that subregion. The base of the first core from Fowey Rocks

(BP-FR-1) was dominated by *Orbicella* spp. rather than *A. palmata*, and because there were significant age-reversals in this section[10] it was not included in this analysis. Additionally, because five *A. palmata* ages in this core from the Early Holocene had significant overlap in the 2σ-ranges of adjacent ages (i.e., they were not statistically different from one another)[10,11], only the oldest and youngest (non-overlapping) *A. palmata* ages from the Early Holocene were used to estimate reef accretion at this location. Similarly, because the bottom-two ages from the second Fowey Rocks core (BP-FR-2) were not significantly different from one another, we only calculated accretion rates from the two non-overlapping *A. palmata* ages for the Early Holocene. We also calculated an accretion rate from the interval 9.4–8.0 ka in this core, but we did not include it in any statistical analysis because the upper age was not from *A. palmata* and it was unclear how much of the interval was deposited during the Early Holocene as opposed to the Middle Holocene. We were able to estimate accretion rates from the top- and bottom-ages of two sequences (B and C) from the OR of Broward[1,2]. The middle ages in those sequences were excluded because they were not significantly different from adjacent ages (Sequence B) or because of a significant age-reversal (Sequence C)[5]. The data from Sequences A and D were not included because the corals in those sequences were likely not *in situ*[6].

The reef framework of the SFCRT was dominated by *A. palmata* during the Early Holocene, when the rates of sea-level rise were most rapid[1,2,5,12]. By the Middle Holocene, however, sea-level rise had slowed, and, as a result, reefs composed of mixed *A. palmata* and massive-coral framework were common[3]. We considered the rates of reef accretion by *A. palmata* and massive-coral or mixed facies separately, using published core logs as a guide[3,10], so that accretion rates of *A. palmata* reefs could be directly compared between the Early and Middle Holocene. We were able to estimate Middle-Holocene reef accretion from one *A. palmata*-dominated sequence

sampled in this study from the Government Cut dredge exposure (PM-25mE)[5]. Again, because many of the nine ages in this sequence were not significantly different from one another we calculated accretion from an age at the bottom of the reef framework (-14.1 m MSL, MD-OR-PM-14.4) and the uppermost sample from that location (MD-OR-PM-10.7b). The majority of our reef-accretion estimates from the Middle Holocene come from 14 intervals with non-overlapping ages from eight of the reef cores collected by Stathakopoulos and Riegl[3] on the IR of central Broward County. A core collected from the same region by Banks et al.[4] (core BR-IR-B-1) provides one additional estimate of accretion of an *A. palmata* reef from the IR. Those researchers also collected the only core from the MR (BR-MR-FL-1), and we used that record to estimate accretion by massive corals in this habitat during the Middle-to-Late Holocene. Finally, we calculated accretion rates from the non-*Acropora* reef framework preserved in the upper layer of each of the cores from Fowey Rocks. We note, however, that accretion on the Fowey Rocks Outlier Reef terminated around the same time as the ORs of the SCRFT[10]. By the Middle Holocene these reefs were in relatively deep water (paleodepths of 7.8 and 10.5 m for BP-FR-1 and BP-FR-2, respectively)[10], the reef was no longer keeping pace with sea level, and accretion was negligible compared with shallow-water reefs in the region. We did not, therefore, include these data in the statistical analyses of reef accretion on the SFCRT.

We compared our data on accretion of reef framework by *A. palmata* to framework built by massive/mixed species from the IR of Broward County to test the hypothesis that rates of reef accretion did not differ between these facies. The data were compared with an independent t-test. The data met the assumptions of normality (Shapiro-Wilk test: $W=0.95$, $p=0.49$) and homogeneity of variances (Levene's test: $F_{1,13}=1.06$, $p=0.32$) after natural-log transformation. We found that although accretion rates of facies dominated by massive corals were somewhat slower, at 2.2 m

ky^{-1} (range=1.1–4.3 m ky^{-1}), than that of *A. palmata* facies, at 3.5 m ky^{-1} (range=1.1–7.5 m ky^{-1}; Table 1, S2), the difference was not significant (t-test: t_{13}=1.34, p=0.200). This result supports the conclusion of several previous studies that although annual growth rates of *A. palmata* are significantly faster than those of massive species, the difference is offset over millennial timescales because of the impacts of processes like reef-framework compaction, bioerosion, and sedimentation[11,13,14].

Climatic correlates of cold-front frequency in south Florida

A number of studies have demonstrated that the frequency of severe winter cold fronts reaching the southern United States is strongly linked to the relative intensity of meridional versus zonal atmospheric circulation over the continent[15-17] (Fig. 5). The association between increased cold-front frequency and intense meridional flow is especially strong in southern Florida, where the occurrence of cold fronts is highly variable from year to year[16,18]. The oscillation between the two modes of atmospheric variability is described by the Pacific North American (PNA) pattern, in which a positive PNA indicates dominance of meridional flow, with increased penetration of winter cold fronts to the south, and a negative PNA indicates more zonal flow and fewer cold fronts[16,19].

As expected, the PNA index has been a strong predictor of recent cold-front frequency in the southern United States in general[15-17], and Florida in particular[16,18]. Indeed, 80% of citrus freezes in Florida between 1899 and 1989 were associated with atmospheric circulation patterns consistent with a positive PNA[18,20]. Furthermore, in the month preceding the winter cold snap of 2010, the coldest winter on record since 1950, there was an abrupt shift in the PNA to the positive phase, which triggered cold air from the northern United States to flow south[15]. The 2010 cold event was also preceded by a shift in the North American Oscillation (NAO), an index of pressure gradients

between the Bermuda High (also called the Azores High) and the Iceland Low in the northern Atlantic, to a negative phase (stronger Iceland Low)[15]. Consequently, there is also a strong association between the NAO index and historic cold-front frequency in south Florida[16-18]. Several studies have evaluated the relationship between cold-front frequency and the El Niño–Southern Oscillation (ENSO); however, whereas some analyses have found a link between cold fronts and El Niño[16,19], the relationship is complex and not always predictable. Other studies found either no relationship between cold fronts and ENSO[16] or that the influence of other climate drivers (i.e., PNA and NAO) were stronger during ENSO-neutral years[17]. Because of the uncertainty related to ENSO as a potential driver of cold-front frequency, we refrain from drawing any conclusions about how changes in ENSO variability during the Holocene may have influenced the millennial-scale changes in the climate of south Florida.

A recently compiled oxygen-isotope record of precipitation from throughout the United States over the Middle to Late Holocene demonstrated that PNA-like oscillations occurred throughout at least the last 8,000 years[19]. Although this record provides a direct proxy for a long-term shift from PNA- to PNA+ by the Late Holocene, shorter-term oscillations in the degree of meridional circulation are less apparent[19]. As a result, we relied on other, high-resolution paleoproxies to infer likely changes in winter cold-front frequency during the Early and Middle Holocene. One such proxy is a record of sea-salt flux to Greenland as recorded in the GISP2 ice core[21]. That study identified five periods of enhanced marine deposition in that record—before 11.3, 8.8–7.8, 6.1–5.0, 3.1–2.4, and 0.6–0 ka—which they interpreted as representing relatively cool periods associated with expansion of the polar vortex and/or enhanced meridional circulation[21]. This record suggests that in addition to millennial-scale shifts in meridional circulation, higher frequency variability may have also played an important role modulating cold-front frequency.

Another potential indicator of the relative degree of meridional flow is the position of the inter-tropical convergence zone (ITCZ). In the modern environment, the ITCZ tracks meridional heat flux, migrating annually between ~2°N in the boreal winter and 9°N in the boreal summer over the Atlantic, eastern Pacific, and central Pacific[22]. Likewise, over longer, millennial timescales, the mean position of the ITCZ largely followed changes in Northern Hemisphere summer insolation, having occupied its northernmost position during the relatively warm climate of the Holocene Thermal Maximum (Fig. S5; HTM), and having moved to the south as climate cooled[22,23]. Liu et al.[19] suggested that the long-term shifts in the position of the ITCZ contributed to PNA-like oscillations during the Holocene, as equatorial migrations of the ITCZ are associated with a southern shift in the position of the polar jet stream over the southeastern United States during the boreal winter[24]. Conversely, when the ITCZ is located at its northernmost position, zonal energy flux increases[25], reducing meridional flow and moving the polar jet stream north[24]. Thus, centennial- to millennial-scale changes in the mean position and variability of the ITCZ should provide a good proxy for shifts in the relative influence of meridional versus zonal circulation and, therefore, the frequency of extreme cold fronts in south Florida during the Holocene.

Periodic development of intense and persistent upwelling-favorable winds during the late spring and early summer can also cause cold anomalies on the SFCRT[26,27]. These winds bring cold, nutrient-rich water with characteristics similar to deep Gulf Stream water masses onto the outer shelf of the SFCRT[28,29]. The resulting temperatures on the outer shelf can be more than 10°C colder than climatological means[30], often resulting in harmful algal blooms and mortality of thermophilic species[31]. These periodic cold-water upwelling events may limit modern coral growth and reef development throughout the SFCRT, especially along the OR[32]; however, because these

events are not predictable over millennial timescales, we were not able to evaluate the possibility that upwelling contributed to reef shutdown on the SFCRT during the Holocene.

Holocene temperature reconstructions

In recent years, there have been a number of efforts to combine the available paleotemperature data into a composite temperature reconstruction for the Holocene[33-36]. The best-known is the reconstruction developed by Marcott et al.[33], which combined 73 records from around the world to create a composite of global temperature change over the last 11.3 ky (Fig. S5). Since its publication, several follow-up studies have emphasized that this 'global' composite is strongly influenced by records from the North Atlantic and may not truly reflect global temperature[33,36,37]. The bias is evident when the global temperature stack of Marcott et al.[33] (Fig. S5, black line) is compared with a reconstruction that excludes records from the North Atlantic (Fig. S5, light gray line), which shows no significant change in temperature over the last 10 ky. In contrast, the Holocene temperature trends reflected in the global curve are even more exaggerated when only Northern Hemisphere records are considered (Fig. S5, dark gray line), suggesting that there are broader-scale climate influences on the global trend.

Although it is clear that the trend from warming during the HTM to cooling in the Late Holocene reflected in the Marcott et al.[33] global temperature composite is largely driven by high-latitude, Northern Hemisphere variability, we chose to focus on this reconstruction in our discussion of changes in mean climate during the Holocene for several reasons. Most importantly, environmental variability in the subtropical habitats of south Florida is closely linked to broader-scale, Northern-Atlantic climate via a variety of oceanographic and atmospheric teleconnections[16,19,38-40]. As a result, mean shifts in the climate of south Florida are likely correlated with extra-tropical (30–60°N) to high-latitude Northern Hemisphere variability, more

so than are the other, more tropical coral-reef ecosystems of the western Atlantic[11,39]. Recently, Kaufman et al.[35] provided an updated, but lower-resolution, series of temperature reconstructions based on 679 records from around the world (Fig. S5, blue lines). The general trends in the Marcott et al.[33] record that inform our Discussion are also represented in the global and extra-tropical Northern Hemisphere composites in the newer reconstruction, supporting our conclusion from Marcott et al.[33] about changes in the mean climate of south Florida.

SUPPLEMENTARY FIGURES

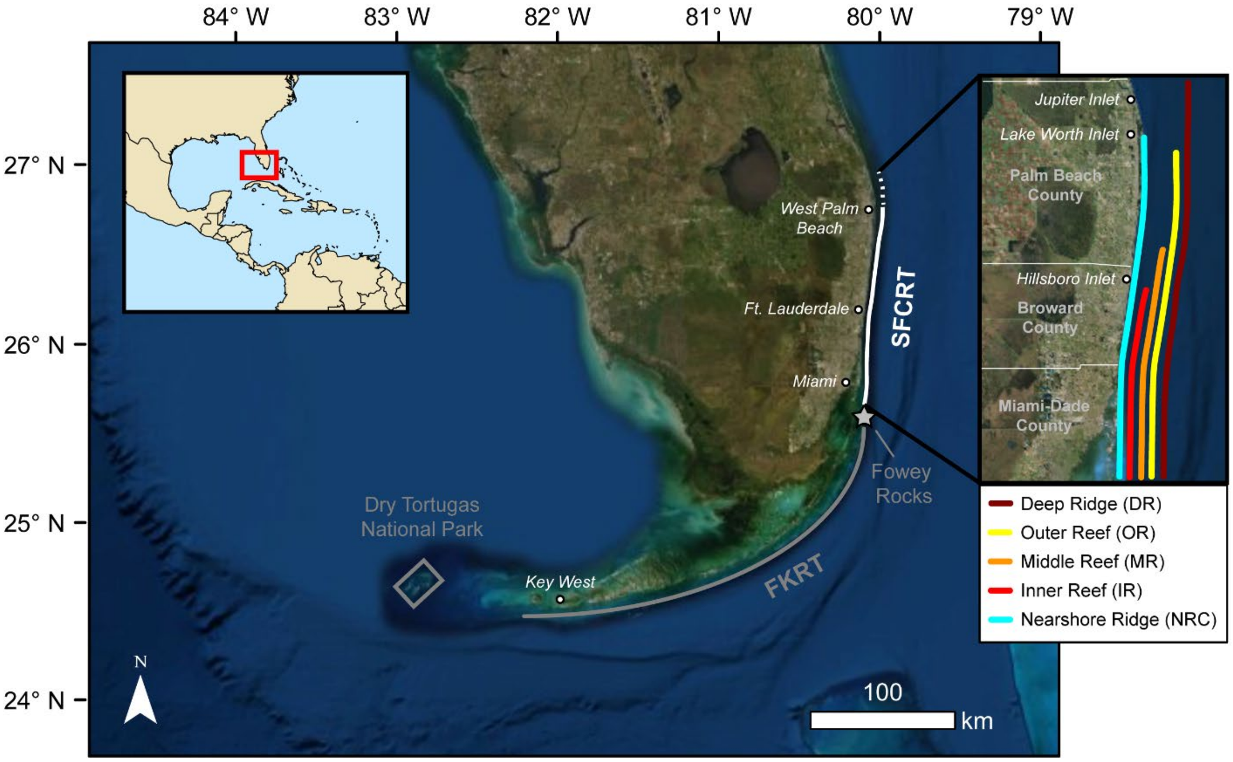

Figure S1. Map showing the location and extent of the Florida Keys Reef Tract (FKRT; gray) and the Southeast Florida Continental Reef Tract (SFCRT; white). The expanded panel on the right shows the extent of the five reef ridges that make up the SFCRT (after Walker[7]). Note that the widths and longitudinal locations of the reef ridges are not to scale. Map image is the intellectual property of Esri and is used herein under license. Copyright 2020 Esri and its licensors. All rights reserved.

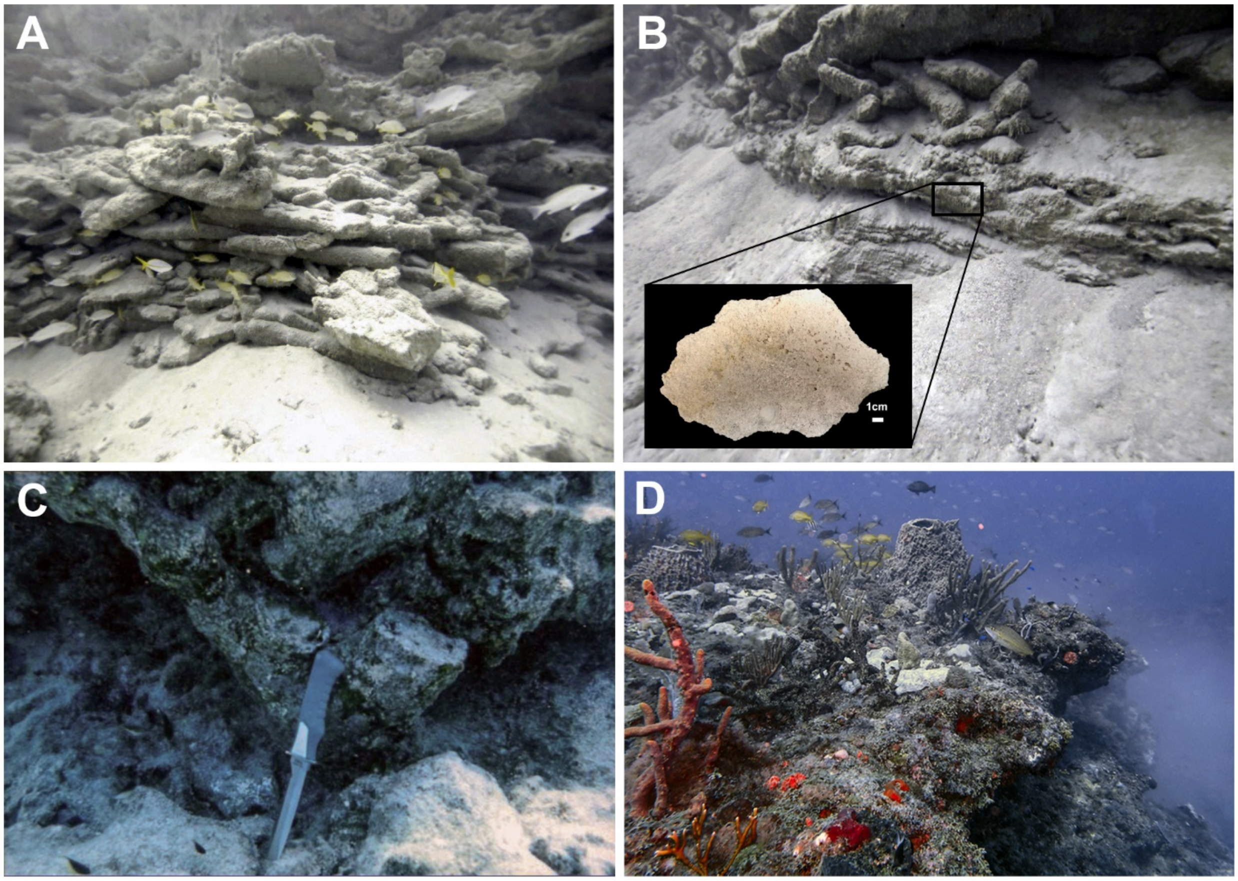

Figure S2. Photographs of Holocene *Acropora palmata* reef framework from locations throughout southeast Florida evaluated in this study. (a) *A. palmata* reef framework of the Outer Reef (OR) in south Miami exposed by dredging associated with the Port Miami Deepening Project at -14.6 m mean sea level (MSL). (b) Holocene beachrock disconformably overlain by *A. palmata*-dominated Holocene reef facies at the base of the OR within the Port Miami dredge exposure at -16.2 m MSL. The beachrock is poorly indurated and dips in a seaward direction (cf. Ginsburg[41]). A sample collected from this deposit (inset photo) shows that the beachrock is composed of mixed siliciclastic (quartz) and carbonate sands. The carbonate sands are composed mostly of fragmented but diagenetically unaltered mollusk and *Halimeda* tests with abundant peloids, characteristic of beachrock deposits[41]. Small vugular birdseye features similar to those described in beachrock deposits by Shinn[42] are also present. Both Port Miami photographs by WFP, 17 September 2014. (c) *A. palmata* reef framework exposed by the grounding of the *USS Memphis* on the IR in Broward. Photograph by WFP, June 2000. (d) Edge of the exposed trench dominated by *A. palmata* framework that we sampled off Palm Beach, FL. Water depths at this location range between -14 and -20 m MSL. Photograph by AEO, 8 July 2019.

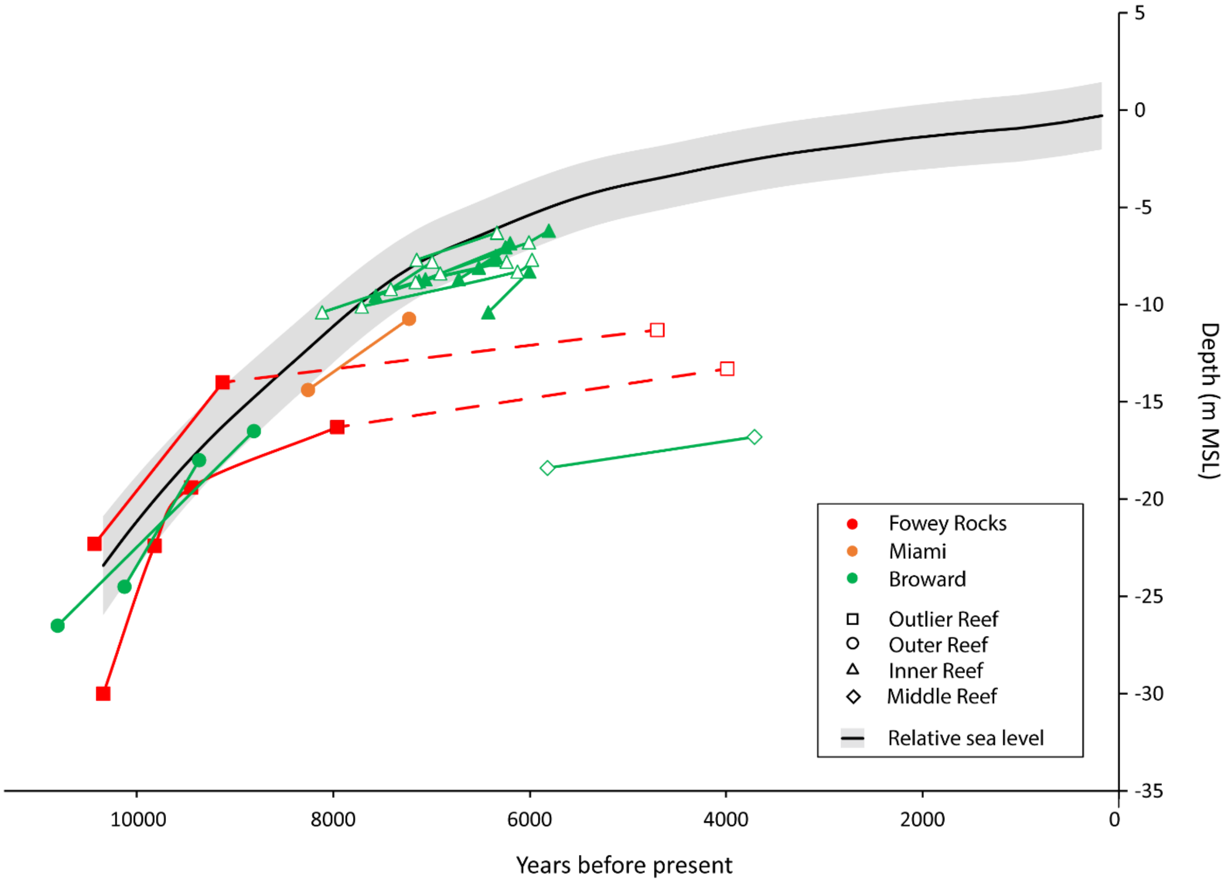

Figure S3. Reef accretion by *Acropora palmata* (closed symbols) and massive/mixed facies (open symbols) on the Southeast Florida Continental Reef Tract in relation to sea level in meters relative to mean sea level[12] (m MSL). Dashed lines associated with the massive-coral facies from Fowey Rocks indicate that reef accretion was already negligible at this site by the Middle Holocene.

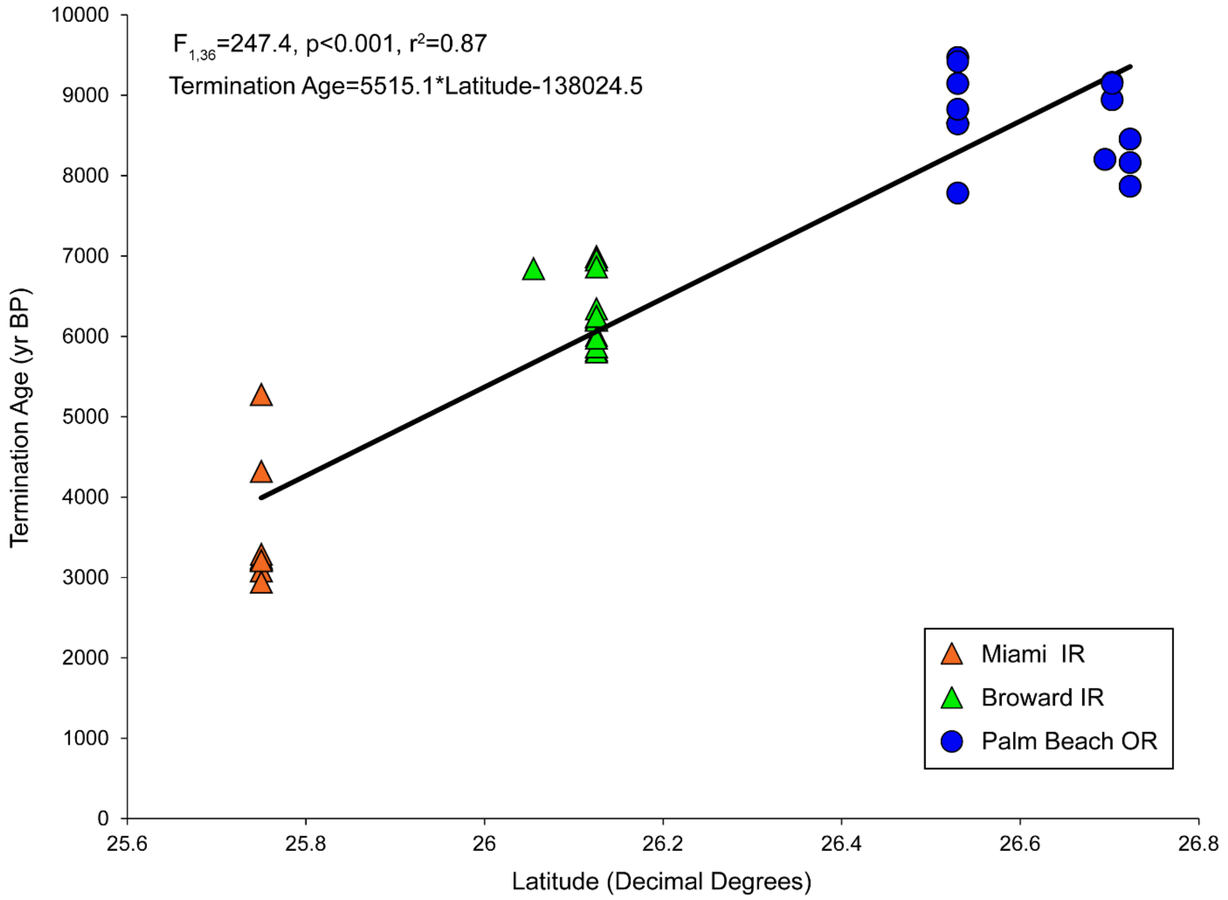

Figure S4. Linear regression of the termination ages (samples collected within 1 m of the reef surface) of *Acropora palmata*-dominated reefs on the Southeast Florida Continental Reef Tract by latitude.

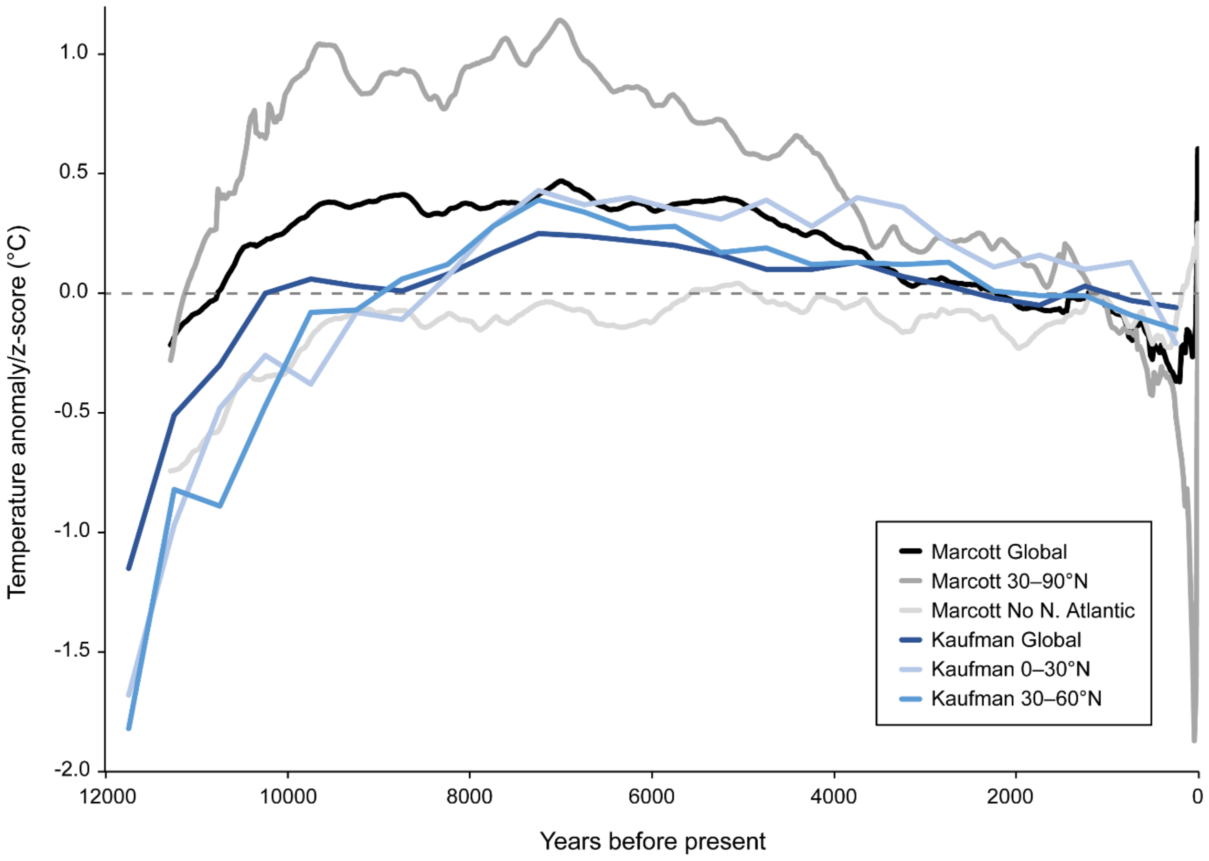

Figure S5. Comparison of the global temperature reconstruction from Marcott et al.[33] (Marcott Global; black line), used as a proxy for mean temperature changes during the Holocene in this study, to other global and regional temperature reconstructions. Two other temperature composites developed in Marcott et al.[33] are included: a composite of records from the Northern Hemisphere extra-tropics, 30–90°N latitude (Marcott 30–90°N; dark gray line), and a global composite that excludes any records from the Northern Atlantic (Marcott No N. Atlantic; light gray line). We also provide three composite records from the most recent Holocene temperature compilation, Kaufman et al.[35]: a global composite (Kaufman Global; dark blue line), a composite of records from the Northern Hemisphere tropics to sub-tropics, 0–30°N latitude (Kaufman 0–30°N; light blue line), and a composite of records from the Northern Hemisphere extra-tropics, 30–60°N latitude (Kaufman 30–60°N; blue line).

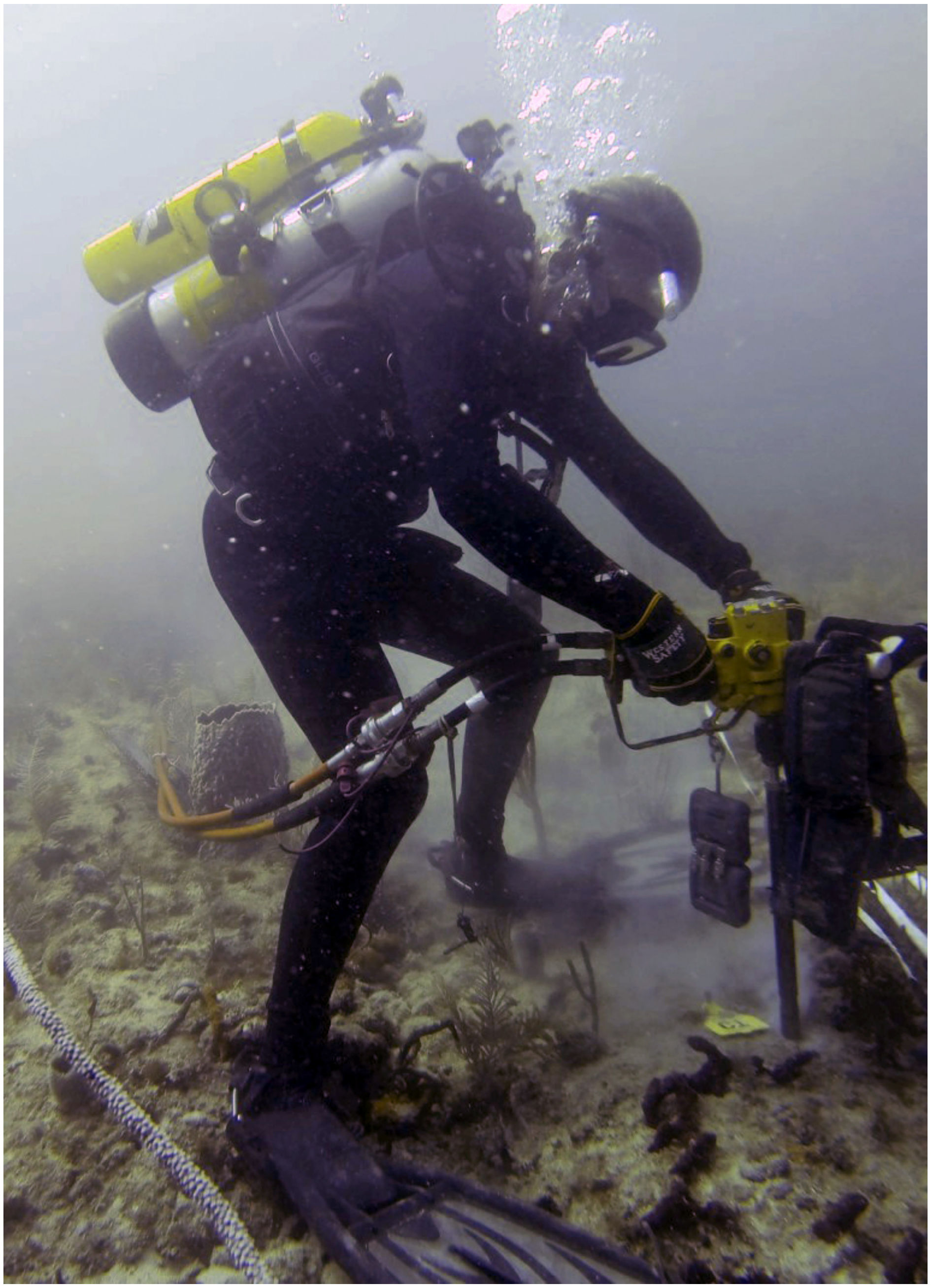

Figure S6. JHH collecting reef-surface cores on the Outer Reef of the southern side of Government Cut in south Miami at -13.1 m mean sea level. Photograph by WFP, September 2013.

SUPPLEMENTARY TABLES

Table S1. Summary of the number of radiometric ages from each of the reef ridges (OR=Outer Reef, MR=Middle Reef, IR=Inner Reef) in each subregion of the Southeast Florida Continental Reef Tract.

Subregion	Reef	Total number of ages	Number of *Acropora palmata* ages	Number of surface *Acropora palmata* ages
Miami	Outlier	15	9	0
	OR	34	29	19
	MR	2	0	0
	IR	12	8	8
Broward	OR	10	10	4
	MR	3	0	0
	IR	51	32	14
Palm Beach	OR	16	16	16

Table S2. Vertical accretion rates of reefs (IR=Inner Reef, MR=Middle Reef) on the Southeast Florida Continental Reef Tract dominated by massive coral framework or mixed *Acropora palmata*–massive-coral facies. Accretion rates are reported in meters per thousand years (m ky^{-1}) for dated reef sequences during the Middle Holocene (see Methods in the main text). Age ranges represent the lower and upper ages of each interval over which accretion rates were calculated. The ranges of elevations of samples used to calculate accretion are given in meters below mean sea level (m bMSL). The accretion rates indicated by asterisks were not included in any statistical analysis.

Period	Subregion	Location	Sequence	Reef	Age range (ky BP)	Elevation range (m bMSL)	Accretion rate (m ky^{-1})
Middle Holocene	Miami	Fowey Rocks	BP-FR-1	Outlier	9.1–4.7	14.1–11.3	0.6*
	Miami	Fowey Rocks	BP-FR-2	Outlier	8.0–4.0	16.3–13.3	0.8*
	Broward	Caves Reef	BR-IR-CR-2	IR	7.2–6.3	7.7–6.3	1.7
	Broward	Caves Reef	BR-IR-CR-3	IR	7.2–6.2	8.9–7.8	1.9
	Broward	Caves Reef	BR-IR-CR-3	IR	7.2–6.2	8.9–7.8	1.9
	Broward	Caves Reef	BR-IR-CR-6	IR	7.4–7.0	9.2–7.8	3.3
	Broward	Caves Reef	BR-IR-CR-9	IR	6.9–6.0	8.4–6.8	1.8
	Broward	Caves Reef	BR-IR-CR-14	IR	7.7–6.1	10.1–8.3	1.1
	Broward	Caves Reef	BR-IR-CR-14	IR	6.1–5.9	8.3–7.7	4.3
	Broward	Middle Reef	BR-MR-FL-1	MR	5.8–3.7	18.4–16.8	0.8

SUPPLEMENTARY REFERENCES

1. Lighty, R. G., Macintyre, I. G. & Stuckenrath, R. *Acropora palmata* reef framework: a reliable indicator of sea level in the western Atlantic for the past 10,000 years. *Coral Reefs* **1**, 125–130 (1982).
2. Lighty, R. G., Macintyre, I. G. & Stuckenrath, R. Submerged early Holocene barrier reef south-east Florida shelf. *Nature* **276**, 59, doi:10.1038/276059a0 (1978).
3. Stathakopoulos, A. & Riegl, B. M. Accretion history of mid-Holocene reefs from the southeast Florida continental reef tract, USA. *Coral Reefs* **34**, 173–187 (2015).
4. Banks, K. W., Riegl, B. M., Shinn, E. A., Piller, W. E. & Dodge, R. E. Geomorphology of the Southeast Florida continental reef tract (Miami-Dade, Broward, and Palm Beach Counties, USA. *Coral Reefs* **26**, 617–633 (2007).
5. Toth, L. T., Precht, W. F., Modys, A. B. & Stathakopoulos, A. Radiometric ages and descriptive data for Holocene corals from southeast Florida. *USGS Data Release*, https://doi.org/10.5066/P9Z21NMU (2021).
6. Stathakopoulos, A., Riegl, B. & Toth, L. T. A revised Holocene coral sea-level database from the Florida reef tract, USA. *Peer J* **8**, e8350, http://doi.org/10.7717/peerj.8350 (2020).
7. Walker, B. K. Spatial analyses of benthic habitats to define coral reef ecosystem regions and potential biogeographic boundaries along a latitudinal gradient. *PLoS One* **7**, e30466 (2012).
8. Shinn, E. A., Hudson, J. H., Halley, R. B. & Lidz, B. H. Topographic control and accumulation rate of some Holocene coral reefs: South Florida and Dry Tortugas. *Proceedings of the 3rd International Coral Reef Symposium* **1**, 1–7 (1977).
9. Lidz, B. H., Reich, C. D. & Shinn, E. A. Regional Quaternary submarine geomorphology in the Florida Keys. *Geological Society of America Bulletin* **115**, 845–866 (2003).
10. Toth, L. T., Kuffner, I. B. & Stathakopoulos, A. Descriptive Core Logs, Core Photographs, Radiocarbon Ages, and Data on Reef Development for Cores of Holocene Reef Framework from the Florida Keys Reef Tract. *USGS Data Release*, https://doi.org/10.5066/F7NV9HJX (2018).
11. Toth, L. T., Kuffner, I. B., Stathakopoulos, A. & Shinn, E. A. A 3,000-year lag between the geological and ecological shutdown of Florida's coral reefs. *Global Change Biology* **24**, 5471–5483, https://doi.org/10.1111/gcb.14389 (2018).
12. Khan, N. S. *et al.* Drivers of Holocene sea-level change in the Caribbean. *Quaternary Science Reviews* **155**, 13–36, https://doi.org/10.1016/j.quascirev.2016.08.032 (2017).
13. Hubbard, D. K. Holocene accretion rates and styles for Caribbean coral reefs: lessons for the past and future. *SEPM Special Publication* **105**, 264–281 (2013).
14. Hubbard, D. K. Depth-related and species-related patterns of Holocene reef accretion in the Caribbean and western Atlantic: a critical assessment of existing models. *International Association of Sedimentologists Special Publication* **41**, 1–18 (2009).
15. Boucek, R. E., Gaiser, E. E., Liu, H. & Rehage, J. S. A review of subtropical community resistance and resilience to extreme cold spells. *Ecosphere* **7**, e01455, https://doi.org/10.1002/ecs2.1455 (2016).
16. Hardy, J. W. & Henderson, K. G. Cold Front Variability in the Southern United States and the Influence of Atmospheric Teleconnection Patterns. *Physical Geography* **24**, 120–137, doi:10.2747/0272-3646.24.2.120 (2003).

17. Hagemeyer, B. C. . ENSO, PNA and NAO scenarios for extreme storminess, rainfall and temperature variability during the Florida dry season. *18th Conference on Climate Variability and Change.* P2.4 (American Meterological Society, Atlanta, 2006).
18. Sheridan, S. C. North American weather-type frequency and teleconnection indices. *International Journal of Climatology* **23**, 27-45, https://doi.org/10.1002/joc.863 (2003).
19. Liu, Z. *et al.* Paired oxygen isotope records reveal modern North American atmospheric dynamics during the Holocene. *Nature Communications* **5**, 3701, doi:10.1038/ncomms4701 (2014).
20. Rogers, J. C. & Rohli, R. V. Florida Citrus Freezes and Polar Anticyclones in the Great Plains. *Journal of Climate* **4**, 1103–1113, doi:10.1175/1520-0442(1991)004<1103:Fcfapa>2.0.Co;2 (1991).
21. O'Brien, S. R. *et al.* Complexity of Holocene Climate as Reconstructed from a Greenland Ice Core. *Science* **270**, 1962–1964, doi:10.1126/science.270.5244.1962 (1995).
22. Schneider, T., Bischoff, T. & Haug, G. H. Migrations and dynamics of the intertropical convergence zone. *Nature* **513**, 45–53, doi:10.1038/nature13636 (2014).
23. Haug, G. H., Hughen, K. A., Sigman, D. M., Peterson, L. C. & Röhl, U. Southward migration of the Intertropical Convergence Zone through the Holocene. *Science* **293**, 1304–1308 (2001).
24. Asmerom, Y., Polyak, V. J. & Burns, S. J. Variable winter moisture in the southwestern United States linked to rapid glacial climate shifts. *Nature Geoscience* **3**, 114–117, doi:10.1038/ngeo754 (2010).
25. Hack, J. J., Schubert, W. H., Stevens, D. E. & Kuo, H.-C. Response of the Hadley Circulation to Convective Forcing in the ITCZ. *Journal of Atmospheric Sciences* **46**, 2957–2973, doi:10.1175/1520-0469(1989)046<2957:Rothct>2.0.Co;2 (1989).
26. Taylor, C. B. & Stewart Jr., H. B. Summer upwelling along the east coast of Florida. *Journal of Geophysical Research (1896–1977)* **64**, 33–40, https://doi.org/10.1029/JZ064i001p00033 (1959).
27. Smith, N. P. Temporal and Spatial Characteristics of Summer Upwelling along Florida's Atlantic Shelf. *Journal of Physical Oceanography* **13**, 1709–1715, doi:10.1175/1520-0485(1983)013<1709:Tascos>2.0.Co;2 (1983).
28. Atkinson, L. P., O'Malley, P. G., Yoder, J. A. & Paffenhöfer, G. A. The effect of summertime shelf break upwelling on nutrient flux in southeastern United States continental shelf waters. *Journal of Marine Research* **42**, 969–993, doi:10.1357/002224084788520756 (1984).
29. Aretxabaleta, A. *et al.* Cold event in the South Atlantic Bight during summer of 2003: Anomalous hydrographic and atmospheric conditions. *Journal of Geophysical Research: Oceans* **111**, https://doi.org/10.1029/2005JC003105 (2006).
30. Pitts, P. A. & Smith, N. P. An investigation of summer upwelling across central Florida's Atlantic coast: the case for wind stress forcing. *Journal of Coastal Research* **13**, 105–110 (1997).
31. Pitcher, G. C., Figueiras, F. G., Hickey, B. M. & Moita, M. T. The physical oceanography of upwelling systems and the development of harmful algal blooms. *Prog Oceanogr* **85**, 5-32, doi:10.1016/j.pocean.2010.02.002 (2010).
32. Precht, W. F. Long-term benthic monitoring of Gulfstream Reef shows “no-effect” of upstream Delray/Boynton wastewater outfall (abstract). *Estuarine Research Federation Biennial Conference.* 171 (Providence, 2007).

33. Marcott, S. A., Shakun, J. D., Clark, P. U. & Mix, A. C. A reconstruction of regional and global temperature for the past 11,300 years. *Science* **339**, 1198–1201 (2013).
34. Mann, M. E. *et al.* Proxy-based reconstructions of hemispheric and global surface temperature variations over the past two millennia. *Proceedings of the National Academy of Sciences* **105**, 13252–13257, doi:10.1073/pnas.0805721105 (2008).
35. Kaufman, D. *et al.* A global database of Holocene paleotemperature records. *Scientific Data* **7**, 115, doi:10.1038/s41597-020-0445-3 (2020).
36. Marsicek, J., Shuman, B. N., Bartlein, P. J., Shafer, S. L. & Brewer, S. Reconciling divergent trends and millennial variations in Holocene temperatures. *Nature* **554**, 92–96, doi:10.1038/nature25464 (2018).
37. Liu, Z. *et al.* The Holocene temperature conundrum. *Proceedings of the National Academy of Sciences* **111**, E3501-E3505, doi:10.1073/pnas.1407229111 (2014).
38. Toth, L. T., Cheng, H., Edwards, R. L., Ashe, E. & Richey, J. N. Millennial-scale variability in the local radiocarbon reservoir age of south Florida during the Holocene. *Quaternary Geochronology* **42**, 130–143, https://doi.org/10.1016/j.quageo.2017.07.005 (2017).
39. Flannery, J. A., Richey, J. N., Thirumalai, K., Poore, R. Z. & DeLong, K. L. Multi-species coral Sr/Ca-based sea-surface temperature reconstruction using *Orbicella faveolata* and *Siderastrea siderea* from the Florida Straits. *Palaeogeography, Palaeoclimatology, Palaeoecology* **466**, 100–109 (2017).
40. Thirumalai, K. *et al.* Pronounced centennial-scale Atlantic Ocean climate variability correlated with Western Hemisphere hydroclimate. *Nature Communications* **9**, 392, doi:10.1038/s41467-018-02846-4 (2018).
41. Ginsburg, R. N. Beachrock in south Florida. *Journal of Sedimentary Research* **23**, 85–92, doi:10.1306/d4269558-2b26-11d7-8648000102c1865d (1953).
42. Shinn, E. A. Practical significance of birdseye structures in carbonate rocks. *Journal of Sedimentary Research* **38**, 215–223, doi:10.1306/74d7191f-2b21-11d7-8648000102c1865d (1968).
